# Marine Invertebrate Metabolites with Anticancer Activities: Solutions to the “Supply Problem”

**DOI:** 10.3390/md14050098

**Published:** 2016-05-21

**Authors:** Nelson G. M. Gomes, Ramesh Dasari, Sunena Chandra, Robert Kiss, Alexander Kornienko

**Affiliations:** 1 REQUIMTE/LAQV, Laboratory of Pharmacognosy, Department of Chemistry, Faculty of Pharmacy, University of Porto, R. Jorge Viterbo Ferreira No. 228, 4050-313 Porto, Portugal; goncalomortagua@hotmail.com; 2 Department of Chemistry and Biochemistry, Texas State University, San Marcos, TX 78666, USA; r_d127@txstate.edu (R.D.); schandra@txstate.edu (S.C.); 3 Laboratoire de Cancérologie et de Toxicologie Expérimentale, Faculté de Pharmacie, Université Libre de Bruxelles, Campus de la Plaine, CP205/1, Boulevard du Triomphe, 1050 Brussels, Belgium; rkiss@ulb.ac.be

**Keywords:** eribulin, trabectedin, mycalamide A, spongistatin 1, stelletin A, monanchocidin A, phenylmethylene hydantoin, frondoside A, discodermolide

## Abstract

Marine invertebrates provide a rich source of metabolites with anticancer activities and several marine-derived agents have been approved for the treatment of cancer. However, the limited supply of promising anticancer metabolites from their natural sources is a major hurdle to their preclinical and clinical development. Thus, the lack of a sustainable large-scale supply has been an important challenge facing chemists and biologists involved in marine-based drug discovery. In the current review we describe the main strategies aimed to overcome the supply problem. These include: marine invertebrate aquaculture, invertebrate and symbiont cell culture, culture-independent strategies, total chemical synthesis, semi-synthesis, and a number of hybrid strategies. We provide examples illustrating the application of these strategies for the supply of marine invertebrate-derived anticancer agents. Finally, we encourage the scientific community to develop scalable methods to obtain selected metabolites, which in the authors’ opinion should be pursued due to their most promising anticancer activities.

## 1. Introduction

The marine environment is by far the major reservoir of biodiversity on the planet Earth and represents the biggest source of the untapped chemical richness that has claimed a considerable attention from the health science communities [1]. Intensive research on the naturally derived metabolites from terrestrial plants and microorganisms as well as their semi-synthetic analogues has led to the approval of several agents that have enriched the anticancer therapeutic arsenal, mainly in the last two decades [2,3,4,5,6,7]. In addition, Herculean efforts from academic research institutions and pharmaceutical companies on the bioprospection of the marine ecosystem have revealed a significant number of lead structures as potential chemotherapeutic candidates, exhibiting promising anticancer effects in *in vitro* and *in vivo* cancer models [8,9,10,11,12]. Furthermore, marine natural products’ usefulness has been also extended to the identification of new molecular targets providing a wider approach for the development of new anticancer agents. Consequently, the dynamic pharmaceutical pipeline comprising several candidates in different stages of clinical development raises hope that several of these candidates may ultimately provide alternative therapeutic tools for cancer treatment [13,14].

Among marine organisms, invertebrates have been the mainstream source in marine-derived drug discovery, contributing approximately to 65% of the marine natural products reported to date, with sponges serving as the most productive source of new anticancer agents in preclinical development [15]. The identification and characterization of the ecological role of complex secondary metabolites produced by marine invertebrates has also led to the discovery of several lead structures displaying relevant pharmacological properties against a wide range of molecular targets [16,17,18]. Linked to the adaptation to the marine ecosystem, these secondary metabolites improve marine invertebrate’s survival by providing chemical defense and adaptation to the marine physical and chemical extreme conditions, compensating their sedentary lifestyle and lack of physical protection [19,20,21,22,23]. Simultaneously, the theoretical concept that these chemical weapons act by interfering with biological receptors and enzymes from co-existing marine competitors and predators, raised the hypothesis that several of those compounds could also interfere with molecular targets involved in carcinogenesis. That hypothesis has been notably supported by the relevant and exciting number of marine invertebrate-derived lead structures with clinical relevance, displaying anticancer properties and targeting tumors with specific and non-specific cytotoxic effects by suppressing several molecular targets [24,25,26,27].

**Figure 1:**
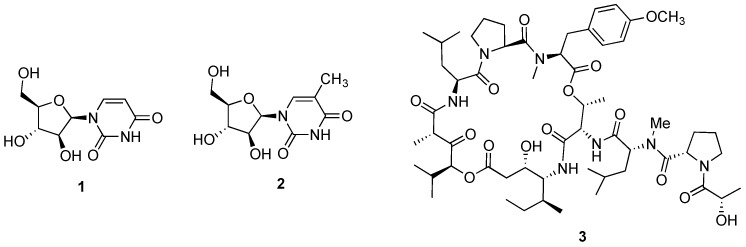
Structures of spongouridine (**1**), spongothymidine (**2**) and didemnin B (**3**).

While the discovery of the nucleosides spongouridine (**1**, Figure 1) and spongothymidine (**2**, Figure 1) from the sponge *Cryptotethya crypta* in the early 1950s by Bergmann and Feeney [28,29], can be considered as the first steps in the development of marine-inspired anticancer agents, it was the cyclic peptide didemnin B (**3**, Figure 1), originally reported from the encrusting ascidian *Trididemnum solidum* [30], that was approved as the first marine naturally occurring candidate to proceed into clinical trials as an antitumor agent. Despite its promising and early successes against several human cancer cell lines, the Phase II clinical trial was terminated in the 1990s by the NCI (National Cancer Institute), due to didemnin B toxicity [31]. However, those preliminary and inspiring efforts along with the improvements in sampling and scuba techniques as well as spectroscopic and spectrometric technologies, encouraged and led to the development of several research programs focused on the bioprospection and characterization of marine-invertebrate derived compounds and assessment of their potential anticancer properties [32,33]. Mainly in the last two decades, the intensive efforts and focus on marine invertebrates as prolific producers of anticancer candidates and the development of synthetic analogues derived from natural prototype structures, keeps exponentially feeding the preclinical and clinical investigation pipeline [13,14,34]. While the majority of these potential candidates exhibiting potent and selective anticancer effects remain in preclinical investigation stages, the current oncological clinical pipeline consists of four marine-inspired anticancer drugs approved by the FDA and EMA, and 18 candidates in clinical trials [35]. Notably, the majority of these compounds correspond to analogues of natural lead structures originally reported as being sourced from a marine invertebrate.

As of the end of 2015, the current marine anticancer pharmaceutical pipeline consists of seven marine-derived approved drugs. Consistent with the major focus on marine invertebrates as sources of pharmacologically active lead structures, it is not surprising that the biggest slice of the anticancer agents so far approved and in clinical development stages are classified as derived from marine invertebrate-derived metabolites, or suspected to be produced by associated microorganisms, as detailed in a following section.

**Figure 2:**
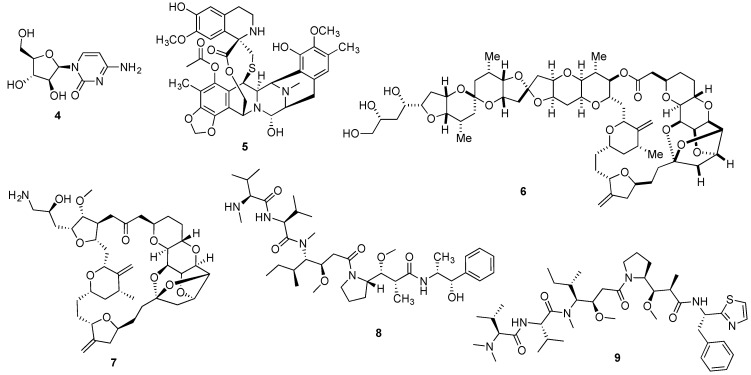
Structures of cytarabine (**4**), trabectedin (**5**), halichondrin B (**6**), eribulin (**7**), monomethylauristatin E (**8**) and dolastatin 10 (**9**).

The first marine-inspired drug to receive FDA approval was cytarabine (**4**, Figure 2), receiving its approval in 1969 for the treatment of acute myelogenous leukemia. The synthetic pyrimidine nucleoside was developed from the spongean nucleoside spongothymidine (**2**, Figure 1). Even nowadays, cytarabine is the mainstream drug for the treatment of acute myelogenous leukemia under the tradename (Cytosar-U^®^), being also used in combination therapy for the treatment of acute lymphoblastic leukemia and chronic myelogenous leukemia [36,37]. More recently, a liposomal formulation (DepoCyt^®^) was approved both by the FDA and EMA for the prevention and treatment of lymphomatous meningitis [38,39]. Furthermore, cytarabine is currently being evaluated against several types of cancer with distinct etiologies, with 278 and 132 active studies being listed in the US and European clinical trials databases, respectively [40,41].

Despite the early reports on trabectedin’s (**5**, Figure 2) remarkable anticancer properties, it was only in 2007 that the antineoplastic marine natural product received market authorization by the EMA under the tradename Yondelis^®^ for the treatment of advanced soft tissue sarcoma and later, for the treatment of recurrent platinum-sensitive ovarian cancer in combination therapy [42]. Very recently, Yondelis^®^ was also approved by the FDA for the treatment of liposarcoma and leiomyosarcoma as the 2nd line therapy [43]. Yondelis^®^ development was significantly delayed due to its low natural abundance and several approaches were applied to overcome the scale-up limitation. Due to its interesting multi-strategy development, a detailed case study on Yondelis^®^ is presented later in this review.

The naturally occurring macrocyclic lactone polyether halichondrin B (**6**, Figure 2), initially reported from the Japanese sponge *Halichondria okadai* in 1985 [44,45], led to the development of eribulin mesylate (Halaven^®^) (**7**, Figure 2). With enhanced anti-tumor activity, the structurally simplified synthetic variation of halichondrin B, retained the tubulin inhibitory properties, being approved by the EMA and FDA to treat locally advanced or metastatic breast cancer, in patients who have received at least two prior chemotherapy regimens for late-stage disease [46,47]. Additionally, Halaven^®^ is also used in the US for the treatment of liposarcoma [47]. At the time of writing, a total of 54 ongoing clinical trials have been listed in European and US databases [48,49].

Approved in 2011 by the FDA and receiving a conditional approval by the EMA in 2012 for the treatment of relapsed or refractory CD30^+^ Hodgkin lymphoma and systemic anaplastic large cell lymphoma, brentuximab vedotin (Adcetris^®^) was the most recent marine-inspired anticancer agent drug to receive market authorization [50,51]. The immunoconjugate Adcetris^®^ consists of the combination of monomethylauristatin E (**8**, Figure 2) linked to an antibody targeting the membrane protein CD30. Monomethylauristatin E corresponds to a semi-synthetic analogue of the linear depsipeptide dolastatin 10 (**9**, Figure 2), originally reported by Pettit’s group from the sea hare *Dolabella auricularia* [52], which reached Phase II trials but was dropped due to associated toxicity and lack of efficacy [53,54].

While the clinical development of several candidates has been stopped predominantly due to lack of efficiency and toxicity, the failure of promising marine-derived anticancer candidates that frequently do not even reach clinical trials has been partially attributed to limitations of the scale up process [6]. The typical long time periods generally involved in the drug discovery process from natural sources, and the difficulties in re-accessing the marine source of the samples due to ecological considerations or even governmental policies, have delayed or even stopped the development of several clinical candidates [55]. Drug development based on the collection from natural populations is also hampered by insufficient and unsustainable quantities of metabolites produced by the animals, often at concentrations below milligrams per kilogram of invertebrate biomass [56], as well as low invertebrate populations [34,57]. While the concentrations of these promising clinical candidates may usually be sufficient for their chemical characterization and preliminary evaluation of the pharmacological profile, they are far from providing a sustainable supply for pre-clinical and clinical development, demanding quantities in a gram range, or future commercialization where annual needs between 1 and 5 kilograms per year are usually estimated [55]. However, several new approaches have been developed aiming to address this major limitation as discussed in the current review.

## 2. Solutions to the “Supply Problem”

### 2.1. Biosynthetic Origin of Marine Invertebrate Metabolites

The recent findings indicating that marine invertebrate-associated microbes may be the true producers of secondary metabolites with potential clinical applications, namely in oncology, can provide viable approaches for the sustainable supply of intermediate or even lead structures. This hypothesis has attracted considerable interest and represents an exciting alternative since, unlike marine animal resources, the supply issue is less problematic for marine microorganisms through animal-independent or even culture-independent production methods [58].

Marine invertebrates as well as plants and fungi have co-evolved with bacterial symbionts, with an expanding list of pharmacologically active natural products being identified in symbiotic bacteria [59]. It is long known that marine invertebrates, mainly sponges, harbor a vast and diverse set of associated microorganisms, many of which are true symbionts. A 16S ribosomal DNA library construction and *in situ* hybridization analysis have allowed the phylogeny of such microbial communities to be uncovered [60,61]. Particularly in sponges, the associated microbiota, mainly extracellularly located, can contribute up to 40%–60% of the animal biomass [62,63]. The co-evolution of marine invertebrates together with endo- and epibiotic microorganisms, that constantly metabolize invertebrate products and synthesize numerous secondary metabolites, points to a chemo-ecological strategy allowing chemical defense, adaptation of the host to the marine environment, and nutrition [64,65,66].

While it is clear that several metabolites are clearly located in an invertebrate tissue and a microbial origin seems improbable, in some cases compounds isolated from sponges appear to be synthesized by their associated microorganisms [67,68]. Despite being less studied than sponges, the microbial diversity associated with bryozoans and ascidians has been also characterized with several secondary metabolites suspected to be produced by symbiotic microbes [69,70,71]. The early suspicions that symbiotic bacteria are the true metabolic sources of several compounds were based on structural homology between some invertebrate-derived compounds from taxonomically distant taxa of marine macroorganisms as well as their resemblance to bacterial natural products [72,73,74,75].

Marine-derived fungi have provided a significant number of candidates with promising anti-cancer properties [76]. However, based on the chemical pattern of secondary metabolites produced by invertebrate-derived fungi, it is doubtful that fungi are metabolic producers of compounds reported from marine invertebrates [76,77]. Instead, several structurally complex polyketides and nonribosomal peptides reported from marine invertebrates, are most likely produced by bacteria, since the biosynthetic machinery responsible for their production, consisting on polyketide synthase (PKS) and nonribosomal peptide synthase (NRPS) pathways, has only been described in bacteria and lower eukaryotes, being extremely rare in animals [74,78].

Several naturally occurring chemotherapeutic candidates reported from marine invertebrates that progressed to pre-clinical or clinical development, are hypothesized to have a microbial biosynthetic source [79]. A representative example is didemnin B (**3**, Figure 1) recently reported as a metabolic product of the *α*-proteobacterium *Tistrella mobilis* collected from marine sediments that while not being conclusive about the metabolic source, indirectly suggests a microbial origin [80]. Also for the lead antitumor agent halichondrin B (**6**, Figure 2), circumstantial evidence points to a microbial source due to its isolation from several classes of Porifera from distinct geographical sites, as well as the structural homology with some bacterial polyethers [81,82]. Stronger evidence exists for the antimitotic agent dolastatin 10 (**9**, Figure 2) isolated from its cyanobacterial diet of the genera *Simploca* and *Lyngbya*, along with several dolastatin-like analogues [83,84].

**Figure 3:**
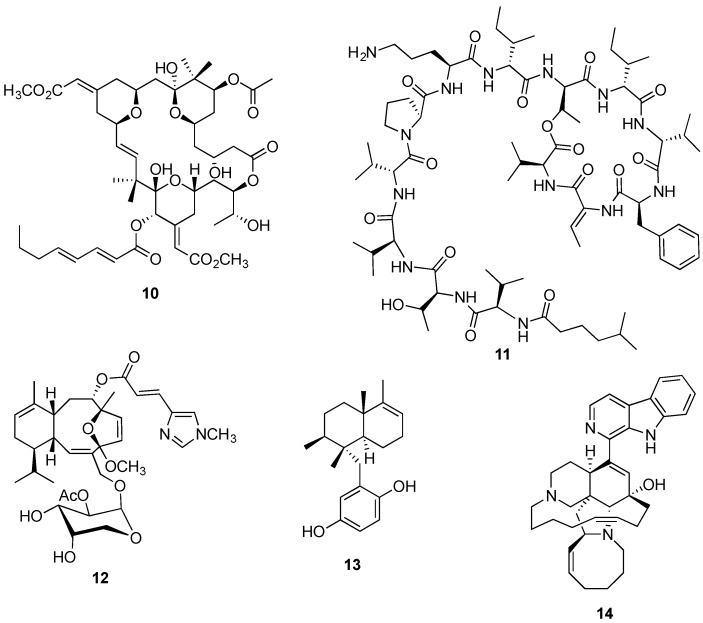
Structures of bryostatin 1 (**10**), kahalalide F (**11**), eleutherobin (**12**), avarol (**13**), and manzamine A (**14**).

Another example is the PKC inhibitor bryostatin 1 (**10**, Figure 3) currently being evaluated in a Phase II trial in Alzheimer patients and listed in several Phase I and II clinical trials against hematological and solid tumor cancers [85,86]. There is almost conclusive evidence that bryostatin 1 is in fact produced by a bacterial symbiont due to the discovery of its putative PKS-I genes, in the genome of the bryozoan symbiont γ-proteobacterium *Candidatus Endobugula sertula* through a metagenomic strategy [87,88]. Further evidence is derived from the observation that *Bugula neritina* colonies treated with antibiotic experienced pronounced decrease on the expression of the PKS gene cluster as well as in bryostatin 1 production [87,89].

The depsipeptide kahalalide F (**11**, Figure 3) reached Phase II trials in Europe in 2004 as an anticancer agent in patients with non-small cell lung cancer stage IIIB (2004-001253-29). Firstly isolated by Hamann and Scheuer [90] from the herbivorous mollusk *Elysia rufescens*, kahalalide F was reported three years later in its algal diet *Bryopsis* sp. [91], suggesting an alternative source. Later, kahalalide F and additional analogues were described from *Bryopsis* and *E. rufescens* associated bacteria [92], providing robust evidence that the metabolic source corresponds to a bacterial symbiont.

So far providing only a limited circumstantial evidence, the isolation of the antimitotic diterpene eleutherobin (**12**, Figure 3) from distinct taxa such as the soft coral *Eleutherobia* sp. [93] and the encrusting coral *Erythropodium caribaeorum* [94], also points to a common symbiotic microorganism as the true producer. Several additional examples of marine animal compounds presumably originated in symbiotic bacteria have been summarized in relevant reviews [71,95,96,97,98].

Despite the vast number of secondary metabolites sharing close structural similarities between an invertebrate-derived and a bacterial compound, this argument constitutes compelling but indirect evidence. The intimate metabolic associations between invertebrate hosts and associated microbes are extremely difficult to unequivocally ascribe the production of the compounds to the host invertebrate, the symbionts or even to a joint biosynthesis [75,99]. In fact, conclusive evidence exists only in a very limited number of cases [98].

Accurate and high resolution mass spectrometry techniques such as MALDI-TOF-imaging are powerful tools allowing the detection of low quantities of compounds and consequently the identification of secondary metabolites in microorganisms and hosts, as well as the taxonomic identification of individual microorganisms based on their mass fingerprints in complex mixed species assemblages [100]. However, the identification of metabolic sources based on compound localization methods such as microanalysis by mass spectrometry, or through classical approaches such as HPLC and NMR analysis, cannot be considered completely reliable since it is plausible to expect that the location of a certain secondary metabolite in a particular cell type can derive from transfer mechanisms between individual cells by export or sequestration mechanisms, and the biosynthesis of metabolic precursors or intermediates can have an invertebrate or microbial origin [101,102]. For the development and selection of sustainable supply tools allowing the large-scale production of those potential clinical candidates, an unequivocal identification of the biosynthetic producer and the distinction between the collected and metabolic source is highly advisable. An unambiguous assignment of the biosynthetic origin from a complex assemblage of marine organisms thus has to originate at the chemical protein or genetic level, and the increasing number of bacterial PKS genes discovered through metagenomics have strengthened the evidence that a notable number of antitumor compounds discovered from invertebrates are in fact bacterial metabolic products [95,97,103].

### 2.2. Marine Invertebrate Aquaculture

While the wild harvest of marine invertebrates is considered plausible for pre-clinical studies, the collection of larger populations for clinical development and commercial production of an eventual clinical candidate is environmentally unsustainable due to insufficient or inaccessible natural populations and the typical low yields of bioactives [56,104]. Even so, halichondrin B (**6**, Figure 2) pre-clinical development was started by harvesting more than one metric ton of the rare sponge *Lyssodendoryx* sp. from natural populations to afford only 310 mg of pure compound [105]. Also, bryostatin 1 (**10**, Figure 3) progressed to Phase I clinical trials through the wild harvest of nearly 13 ton of the bryozoan *Bugula neritina* yielding 18 g of the anticancer candidate [106].

As an alternative to wild harvesting, aquaculture may represent a plausible strategy in a few cases mainly through *in situ* (mariculture) or sea-based farming, with some successful cases leading to improved growth rates compared to the ones reported from natural populations [107,108]. As an example, the sea-based aquaculture trial of the sponge *Mycale hentscheli*, reported to produce the microtubule-stabilizing agent peloruside A, displayed an impressive growth rate of 3000% in eight months [109], with an estimated supply of one gram of peloruside A per 100 kilograms of sponge biomass [110]. With a disappointingly lower yield, the mariculture of another microtubule-stabilizing agent, eleutherobin (**12**, Figure 3), led only to the isolation of 12 g per ton of the gorgonian *Erythropodium caribaeorum* [111]. The sea farming is clearly dependent on the *in situ* conditions posing serious limitations, such as the difficulty in controlling culture parameters and susceptibility not only to environmental changes but also pathogens [112,113,114]. Additionally, environmental changes can also lead to a distinct associated microbial consortium, which may be critical for metabolites produced by bacterial symbionts [109].

Intensive efforts have also been made in the optimization of *ex situ* or land-based invertebrate’s culture, mainly driven by the possibility of better controlling the farming conditions and optimization of metabolite production [115]. However, despite the potential to obtain a continuous annual growth and avoid disease outbreaks, with a few exceptions [116,117,118,119], *ex situ* farming has so far failed to produce a consistent biomass yield, since it is difficult to mimic the complexity of the invertebrate´s natural habitat. While food quantity and quality requirements are indicated as the primary factors for significant growth rates [120], species-specific conditions, such as water pH, salinity, temperature levels, light exposure, and dissolved oxygen, all need to be controlled for feasible commercial farming [113,114]. Furthermore, it is obviously impossible to assure an efficient feeding supply and waste removal in a closed system as compared to the unlimited volumes of seawater in the natural habitats [112].

The production of secondary metabolites by marine invertebrates is generally quite variable, depending on several physical, chemical, and biological induction factors [114]. Thus, even with an optimal invertebrate growth rate, due to the lack of knowledge of the induction factors it is not certain that a specific bioactive compound will be produced in an unnatural and controlled environment. In fact, as referred by Koopmans *et al.* [121] several examples support a possible activation of metabolic pathways in sponges, which are derived from chemical and physical aggression factors present in an unnatural environment.

While in certain cases, such as the production of up to three g of the antipsoriatic and potent cytostatic agent avarol (**13**, Figure 3) per kilogram of sponge *Dysidea avara* wet weight [122], invertebrate farming appears to be a plausible supply strategy, most species are slow-growing and, in the majority of the cases, the production of the desired metabolite is low as demonstrated with halichondrin B and bryostatin 1. Based on many marine sponge aquaculture trials, it is now generally agreed that sea farming appears to be more successful than *ex situ*. Despite its limitations, such as the vulnerability to destruction by climatic and environmental events, risk of infection and costs associated with metabolite extraction, sea-based aquaculture gives faster invertebrate growth rates and higher yields of metabolite production [114,121,123].

### 2.3. Invertebrate and Symbiont Cell Culture

While whole-animal culture has been used for years as the main supply strategy, in cases where the desired compound is produced by the marine invertebrate, it is also plausible to consider cell culture. The controlled and continuous growth of a specific type of invertebrate cell in bioreactors is a tempting alternative. However, with the main focus on sponge cell culture, only modest progress has been achieved to date [124,125].

Since the majority of sponge cells are totipotent, it has been attempted to obtain homogeneous sponge cell populations that have the ability to produce a specific metabolite *in vitro*. However, a continuous cell growth of primary cells has not been achieved [113]. Recently, primmorphs, a specific type of cells produced from primary cells in suspension, attracted considerable interest. Notably, it was observed that primmorphs formed in the presence of symbionts, leading to the production of a certain metabolite, even if the latter has a microbial origin [126].

Analogously to the *ex situ* aquaculture of marine sponges, it is a Herculean task to not only simulate their natural habitat, but also the cells’ own micro-environment, which may cripple cell growth, and ultimately secondary metabolite production [127]. Additionally, it is clear that the promotion of sponge cell culture growth is also dependent on several specific growth factors and inorganic demands that should be supplemented through specific substrates, in addition to the very specific and strict control of physical parameters [125,128]. Furthermore, for the specific production of potential anticancer secondary metabolites usually displaying cytotoxic effects, cell culture via cell suspension or primmorphs, appears not to be a recommended approach due to the interference on the growth of sponge cells. Notably, Müller’s promising studies led to the successful *in vitro* production of avarol (**13**, Figure 3) from the primmorphs of the sponge *Dysidea avara* [129]. Part of the success may be explained by the avarol’s cytostatic rather than cytotoxic properties, which do not induce cell death of the sponge primmorphs [129].

In their economic analysis of the supply of marine sponge-derived metabolites *via* sponge aquaculture or sponge primmorphs, Sipkema *et al.* [123] clearly indicate that to establish a viable large-scale supply of secondary metabolites through sponge cell culture, the formation of primmorphs would require a significantly higher biomass of animal compared to the required biomass for the direct extraction of a target compound from the sponge. Since the number of variables that interfere with invertebrate cell growth and the required factors that allow for their *in vitro* culture are still vastly unknown, it is clear that, despite the exciting progress achieved to date, the adaptation of this strategy to the large-scale production of invertebrate metabolites still remains a mirage.

As previously mentioned, the detection and/or isolation of secondary metabolites from microorganisms, namely bacterial endosymbionts made marine microorganism culture-dependent approaches a promising supply strategy. Fermentation still plays a key role in the drug development of pharmaceuticals, and the possibility of obtaining a sustainable and economically feasible supply based on microbial large-scale cultures, frequently improves the chances for a potential clinical candidate to progress to clinical trials [75]. Another advantage of *in vitro* microbial culture methods is the possibility of manipulating different growth parameters to increase the yield of secondary metabolites [130].

The identification of several strains of marine-derived microorganisms isolated from marine invertebrates as well as their use to furnish pharmacologically active compounds has been successfully demonstrated through cultivation-dependent approaches [131,132,133,134,135]. However, these strains are almost exclusively surface-associated microorganisms and usually marine facultative [130]. In fact, while most microbial-derived pharmaceuticals are known from culturable strains, genomic studies have revealed that an estimated 99% of both terrestrial and marine bacteria remain uncultured through standard laboratory methods [136,137]. The slow progress on the development of culture-dependent approaches for the production of marine microorganisms, particularly obligate marine bacteria and/or endosymbionts, and the lack of appropriate literature on the isolation procedures and standardized culture conditions, will certainly continue to contribute to this undesirable scenario [34,98].

It is possible that in near future, the majority of the bacterial symbionts associated with marine invertebrates will still remain unculturable *ex hospite* due to host-specific nutrients, cell-cell interactions, and additional not yet defined metabolic factors that are required for growth [138]. While it is unachievable to simulate all the specificities of the marine environment, including the interaction with an invertebrate host or other microbes, several successful attempts have been made to improve the culturability of marine microorganisms based on the simulation of their natural environment [139]. The control of physical cultivation conditions such as temperature or aeration, or media composition and incubation time have been taken into account to assure or even maximize the production of a certain compound, and several successful approaches have been already fruitful [139,140,141,142,143]. However, even if hypothetically the optimization of physical cultivation conditions can be achieved allowing a controlled and significant growth of a marine symbiont, the target compound might not be produced due to the absence of the required environmental signals [78].

Despite the abovementioned limiting factors, the *in vitro* production of a compound by a bacterial symbiont has been achieved by Hill’s group. Successful examples are represented by the production of kahalalide F (**11**, Figure 3) obtained from the cultures of bacteria *Vibrio mediterranei* [92], and the isolation of the antimetastatic alkaloid manzamine A (**14**, Figure 3) from the culture of Gram-positive bacterium *Micromonospora* M42 obtained from the deep-water Indonesian sponge *Acanthostrongylophora* sp. [144].

### 2.4. Culture-Independent Strategies

The privileged access to the marine bacterial biosynthetic machinery through genome mining has led to the identification of a few putative biosynthetic PKS and NRPS genes responsible for the production of anticancer lead structures originally described from marine invertebrates [77,145]. In addition to the chemo-ecological relevance, the recent rapid advances in microbial genomics allow the access and expression of single genes or entire genetic pathways in suitable heterologous hosts paving the way for the sustainable supply of lead structures produced by unculturable or slow-growing bacterial symbionts [78,146,147,148].

The extraction of DNA suitable for heterologous expression from invertebrate bacterial endosymbionts is extremely challenging due to the tight junctions formed. However, this limitation can often be overcome with metagenomics involving the analysis of eDNA from complex populations of organisms as well as mainstream bioinformatics tools [145,149,150,151]. The identification of entire biosynthetic genes in bacterial symbionts is simplified by the organization of bacterial genes in compact clusters facilitating their mining and cloning [78]. To fully explore the potential of heterologous expression, it is indispensable to develop suitable culturable hosts able to efficiently express the natural products from a taxonomically distant organism [152]. Despite the challenges, several strains of *Escherichia coli*, *Pseudomonas putida*, *Streptomyces*, among others have been engineered as amenable hosts to successfully express PKSs and NRPSs from a distinct origin [153,154,155,156,157,158].

**Figure 4:**
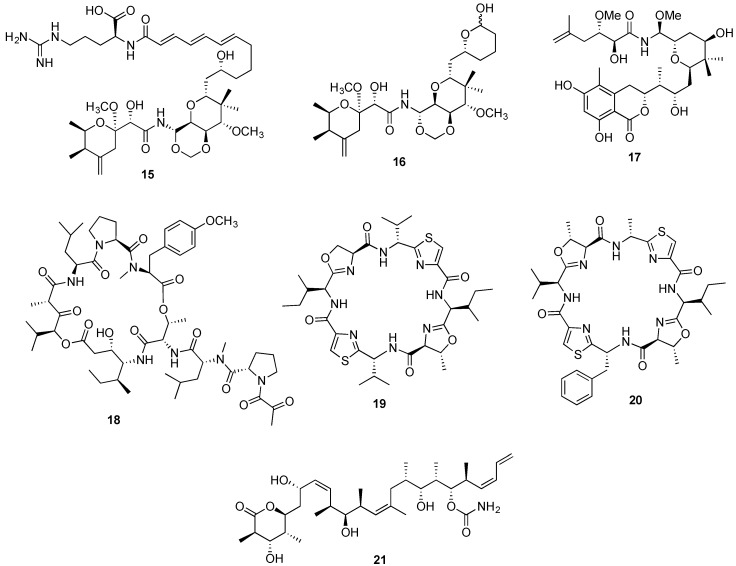
Structures of onnamide A (**15**), theopederin A (**16**) and irciniastatin A (**17**), aplidine (**18**), patellamide A (**19**) and C (**20**), discodermolide (**21**).

Several gene clusters have been identified from bacterial symbionts, predominantly *trans*-acyltransferase PKSs (trans-AT-PKS). Pederin putative trans-AT-PKS cluster was the first identified cluster from an uncultured *Pseudomonas* sp. symbiont of a terrestrial beetle [159]. Motivated by the striking structural similarities between pederin and the spongean antitumor agents onnamide A (**15**, Figure 4), theopederin A (**16**, Figure 4) and irciniastatin A (**17**, Figure 4), Piel and colleagues constructed a metagenomic cosmid library from the DNA of the sponge *Theonella swinhoei*, and through a clone pooling strategy a gene cluster closely resembling pederin trans-AT-PKS cluster was identified, thus providing robust evidence on the bacterial origin of the sponge derived metabolites [160]. Additional examples refer to the identification of bryostatin PKS gene cluster in the genome of a bryozoan bacterial symbiont as previously mentioned [87,88], and the identification of didemnin B gene cluster in the *α*-proteobacteria *Tistrella mobilis* obtained from marine sediments. This suggests that an alternative supply may be achieved for the anticancer clinical candidate aplidine (**18**, Figure 4) through the bacterial production system [80,161].

However, the landmark achievement in the production of marine bacterial symbiont-derived metabolites was the successful “shotgun” cloning of the gene cluster encoding a microcin-like ribosomal pathway responsible for the synthesis of the anticancer peptides patellamide A (**19**, Figure 4) and C (**20**, Figure 4). The sequencing of the entire genome from *Lissoclinum patella* cyanobacterial symbiont *Prochloron didemni*, allowed the patellamide gene cluster in *Escherichia coli* to be expressed through the heterologous expression, providing conclusive evidence on the patellamides’ true metabolic source [162,163].

Notably, in addition to serving as an alternative supply tool for unculturable marine microbes, metagenomics uncovers also the existence of silent gene clusters, constituting a relevant proportion of the microbial genome usually inactive through culture-dependent methods. Allowing their expression may give access to the full metabolic potential of unculturable bacteria making it possible to discover, isolate, and produce new secondary metabolites, potentially with relevant anticancer properties [58,100,164,165]. Still, despite the exciting results obtained through metagenomics, the scale up production of a compound through the heterologous expression methods remains a challenge and their definitive affirmation as feasible and efficient solutions to deliver sufficient quantities for clinical development and subsequent commercialization is still lacking.

### 2.5. Total Chemical Synthesis

Prior to the 20th century, pharmacologically useful molecules were primarily obtained from their natural sources. With the tremendous advances in the development of synthetic methodology and our ability to synthesize more and more challenging molecules, this paradigm has changed and synthesis has become an integral part of not only providing access to the bioactive natural products, but also their derivatives with improved pharmacological properties. Today, the main consideration in developing a synthesis of a clinical candidate is not the ability to reach the desired target, but rather the scalability of the synthetic sequence and the amount of effort, manpower, and resources this undertaking involves. A case in point, is the clinical development of discodermolide (**21**, Figure 4), a polyketide natural product that was isolated from Caribbean deep-sea sponge *Discodermia dissoluta* [166]. Discodermolide displayed significant antitumor activity in preclinical evaluation, but the progression of this natural product toward the clinical development was hampered by the supply problem. Its occurrence constitutes only 0.002 wt % of the dried *D. dissoluta*, and this rare natural source could not provide sufficient quantities of material required for clinical trials. Inspired by the total synthesis of discodermolide by the Paterson and Smith groups [167,168], Novartis Pharma AG developed a 39-step synthesis (26 steps in the longest linear sequence) leading to 60 grams of discodermolide in 2004 [169,170,171,172]. The synthesis took 20 months to complete, involved 43 chemists, and produced the final product in an overall yield of 0.65%, indicating the huge investment of time, manpower, and resources. However, the provision of 60 grams of discodermolide from the natural source would have required 3000 kg of the sponge, an amount which probably does not exist. This accomplishment demonstrates that given enough time, resources, and manpower, total synthesis is capable of delivering sufficient material for clinical studies.

**Figure 5:**
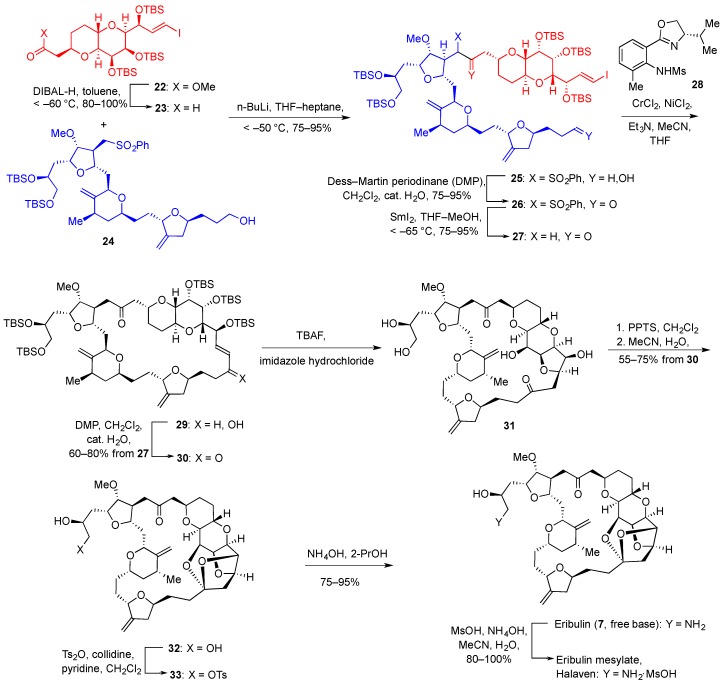
Total synthesis of Halaven^®^ starting from building blocks **22** and **24**.

Of the clinically approved marine invertebrate-derived anticancer drugs, eribulin mesylate (Halaven^®^) is manufactured by total synthesis, developed by Eisai Inc. As mentioned in the Introduction, eribulin is a fully synthetic analogue of halichondrin B and it has been approved for the treatment of metastatic breast cancer. The structure of eribulin (**7**, Figure 2) is significantly simplified compared with halichondrin B (**6**, Figure 2), but it still contains 19 of the 32 stereocenters present in the natural product. The foundation for the developed synthesis of eribulin was the total synthesis of halichondrin B reported by Kishi and coworkers at Harvard University [173]. Shown here is the endgame of the developed synthetic pathway involving the joining of building blocks **22** and **24**, prepared in 13 (2.2% yield) and 18 steps (0.7% yield), correspondingly, from commercial materials (Figure 5).

DIBAL reduction of the ester functionality in **22** led to aldehyde **23**, which was subjected to an aldehyde-sulfone coupling using BuLi in THF-heptane to produce **25** incorporating the entire carbon skeleton of eribulin. Oxidation of **25** with Dess-Martin periodinane followed by the selective removal of the sulfone moiety with samarium(II) iodide gave ketone **27**. The macrocyclic ring closure was accomplished by the Ni(II)/Cr(II)-mediated coupling (Nozaki-Hiyama-Kishi reaction; NHK) to produce **29**. The employment of the asymmetric ligand **28** in this coupling reaction had no stereochemical consequences as the resulting secondary alcohol was oxidized in the next step to a ketone, however it produced the highest reaction rates of the many ligands tested. Ketone **30** was then treated with TBAF to remove the protecting silyl groups and the required ketalization was achieved with PPTS in CH_2_Cl_2_ followed by crystallization from acetonitrile and water to give **32**. The introduction of tosylate at the primary hydroxyl was followed by the treatment with alcoholic ammonium hydroxide. This resulted in the intermediate formation of an epoxide, which underwent ring opening with ammonia to give eribulin free base **7**. The free base was dissolved in acetonitrile and treated with ammonium mesylate, the solvent was replaced with CH_2_Cl_2_ and the obtained salt was precipitated with pentane. After drying *in vacuo*, the resulting eribulin mesylate (Halaven^®^) was obtained as an amorphous powder that was suitable for intravenous formulation and administration.

The developed synthesis of eribulin mesylate is reproducible on a multi kilogram scale and is currently used for the commercial manufacture of this anticancer agent for human clinic. However, the length of the synthesis, amounting to 30 steps for the longest linear sequence, and low overall yield of 0.16% testifies to the investment of resources for the industrial manufacture of this cancer drug.

### 2.6. Semisynthesis

Due to the resources involved in the development of industrial scale manufacture of pharmaceuticals by total synthesis, semisynthesis involving an elaboration of a structurally related natural products to produce the desired one can be an important consideration. Of the clinically approved marine invertebrate-derived anticancer drugs, trabectedin is produced through such an approach.

Trabectedin (**5**, Figure 2, Ecteinascidin 743, Et-743) was first isolated in 1990 by Reinhart and coworkers from the Caribbean tunicate *Ecteinascidia turbinata* as the most abundant representative of a group of six ecteinascidins: Et-729, Et-743, Et-745, Et-759A, and Et-759B [174,175,176,177]. Trabectedin was tested in the National Cancer Institute (NCI) 60-cell line panel and found to have potent antiproliferative activity and mechanism of action distinct from the standard agents in the NCI database as revealed by the COMPARE analysis. Although extensive evaluation of trabectedin in murine models of human cancer revealed broad activity against xenografts derived from a diverse spectrum of tumors [178,179], human phase I clinical trials demonstrated prolonged disease stabilization in soft tissue sarcoma patients [180,181,182]. Subsequently, after trabectedin’s efficacy in soft tissue sarcoma patients was confirmed in phase II clinical trials, it was approved in 2007 in the European Union (Yondelis^®^) for the treatment of advanced or metastatic soft tissue sarcoma after failure of anthracyclines and isofosfamide [183]. Further, after a randomized phase III trial of trabectedin in combination with pegylated liposomal doxoribucin (PLD) demonstrating a significant improvement in progression-free survival and overall response over PLD alone [184,185], trabectedin/PLD combination received approval for the treatment of relapsed platinum-sensitive ovarian cancer in the European Union in 2009 [186]. Most recently, in October 2015, following the results of a randomized phase III clinical trials demonstrating better outcomes in either liposarcoma or leiomyosarcoma patients treated with trabectedin compared to those receiving dacarbazine [187], the US FDA announced the approval of trabectedin for the treatment of liposarcoma or leiomyosarcoma that is either unresectable or has metastasized [188]. Since Yondelis’ approval in the European Union in 2007, close to 50,000 patients in 80 countries have benefited from this therapy and its approval in the US will further help address this unmet clinical need [188].

Extensive preclinical and clinical evaluation of trabectedin followed by its marketing as a cancer drug has led to the supply problem. Generally, marine natural products not only are present in small amounts in the producing organism and also possess highly complex structures. Trabectedin, isolated in 0.0001% yield and possessing bridged multicyclic stereochemically complex structure serves as an excellent example of problems involved in the provision of sufficient amounts of material for its development and use as a cancer drug. Initially, bulk collections of the Caribbean tunicate populations and their extraction followed by purification of trabectedin allowed for provision of the necessary quantities of material to complete *in vitro* studies and initiate preclinical evaluation [189].

However, due to the environmental and sustainability considerations of harvesting large amounts of natural populations [190], PharmaMar, the company that performed the pharmacological evaluation and was responsible for the ultimate commercialization of trabectedin, initiated a challenging program of *Ecteinascidia turbinata* aquaculture and, subsequently, Mediterranean aqua pharms located in the Balearic Islands, Tunisia, and the Atlantic coast of Spain were established. After several years of effort PharmaMar produced over 250 metric tons of the tunicate biomass with the trabectedin content amounting to 5 milligrams per gram on the wet weight basis. However, the isolation of trabectedin involved complex extraction with several different organic solvents followed by multi-step chromatography. This led to final yields of 1 milligram per gram. This low yield of the isolated material combined with the heavy economic impact of the extraction and purification processes highlighted the inability of the aquaculture method to solve the trabectedin supply problem [189]. Indeed, while these efforts provided sufficient quantities of the material to continue clinical development, the prospect of future commercialization of trabectedin necessitated the departure from the dependence on the natural source and called for the development of a scalable synthetic process.

**Figure 6:**
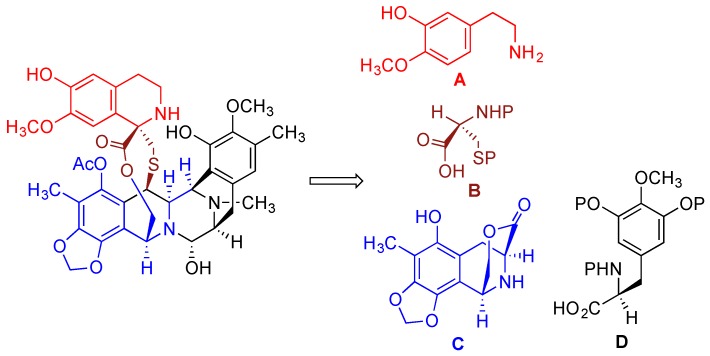
Retrosynthetic scheme illustrating the convergent approach in Corey’s synthesis of trabectedin.

The first total synthesis of trabectedin was accomplished by Corey’s group [191,192]. It was based on the proposed biosynthetic route [193,194] that involved a convergent approach consisting of combining four subunits **A**, **B**, **C,** and **D** depicted in Figure 6 [189,195,196].

**Figure 7:**
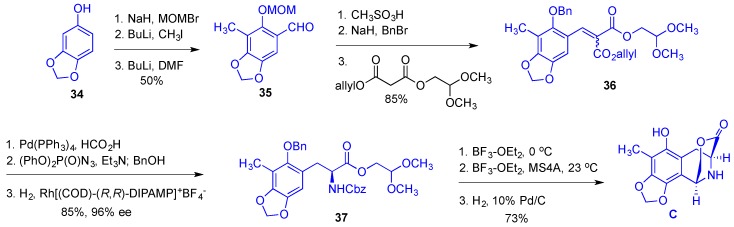
Synthesis of the building block **C**.

Synthesis of bridged lactone **C** commenced with a double *ortho*-derivatization of MOM ether-protected sesamol **34** giving tetra-substituted benzaldehyde **35** (Figure 7). Removal of the MOM group with the subsequent reprotection of the phenolic oxygen as benzyl ether was followed by the Knoevenagel condensation with a double malonate ester to give α,β-unsaturated derivative **36**. Allyl ester in **36** was removed under hydrogenolytic conditions and the resulting acid was subjected to the Curtius rearrangement with the subsequent enantioselective alkene hydrogenation to give Cbz-protected aminoester **37** in 96% ee. Dimethyl acetal was removed with BF_3_ etherate and the presence of water and the resulting aldehyde was subjected to the treatment with BF_3_ etherate and MS 4A effecting an intramolecular Pictet-Spengler cyclization, which after the removal of Bn and Cbz groups afforded the desired lactone **C**. The synthetic sequence leading to the building block C consisted of 12 steps giving the desired product in 26% overall yield.

**Figure 8:**
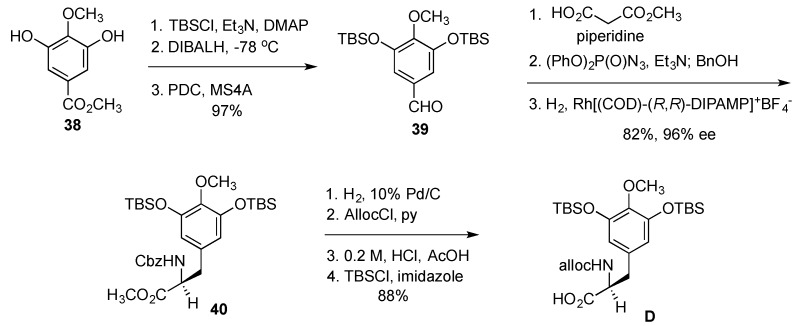
Synthesis of the building block **D**.

Synthesis of the building block **D** started with gallic ester **38**, which was converted to benzaldehyde **39** with routine transformations (Figure 8). Knoevenagel condensation with monomethylmalonate, incorporation of nitrogen with Curtius rearrangement and asymmetric hydrogenation of the alkene gave Cbz-protected tyrosine derivative **40**. Changing the protecting group on the nitrogen from Cbz to Alloc, methyl ester hydrolysis and the reinstallment of the TBS ethers gave the desired building block **D** in 10 steps and 70% overall yield from gallate **38**.

**Figure 9:**
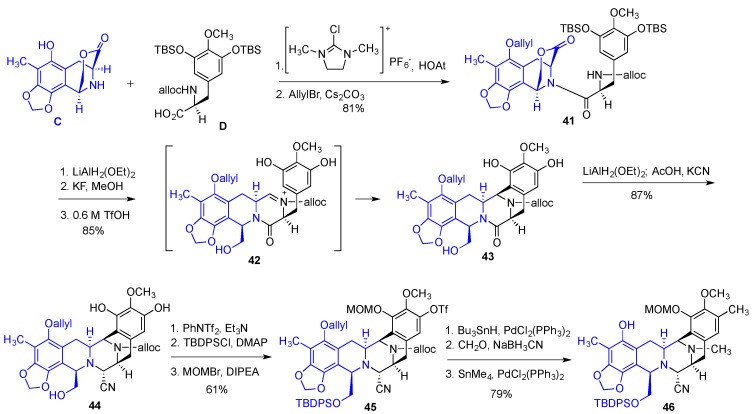
Linking building blocks **C** and **D** and further manipulations to prepare for the incorporation of fragments **A** and **B**.

The two building blocks **C** and **D** were combined by the amide formation reaction promoted with 2-chloro-1,3-dimethylimidazolidinium hexafluorophosphate (CIP) and 1-hydroxy-7-azabenzotriazole (HOAt), affording after allylation of the phenolic oxygen amide **41** (Figure 9). Reduction of the lactone functionality in **41** to the corresponding lactol, removal of the TBS ethers and treatment with triflic acid affected the desired bisannulation to give **43** through the intermediacy of acyliminium ion **42**. Partial reduction of the amide in **43** to the aminal derivative was followed by treatment with KCN in AcOH to afford aminonitrile **44**. This was followed by regioselective triflation of one of the two phenolic hydroxyls, silylation of the primary alcohol, and introduction of the MOM group on the remaining phenolic oxygen to give **45**. The allyl ether in **45** was hydrogenolytically removed and the two necessary methyl groups were introduced through reductive amination and Stille coupling resulting in intermediate **46**, possessing the necessary functionality for the incorporation of the remaining building blocks **A** and **B**. Synthesis of **46** from fragments **C** and **D** proceeded in 13 steps and 29% overall yield.

**Figure 10:**
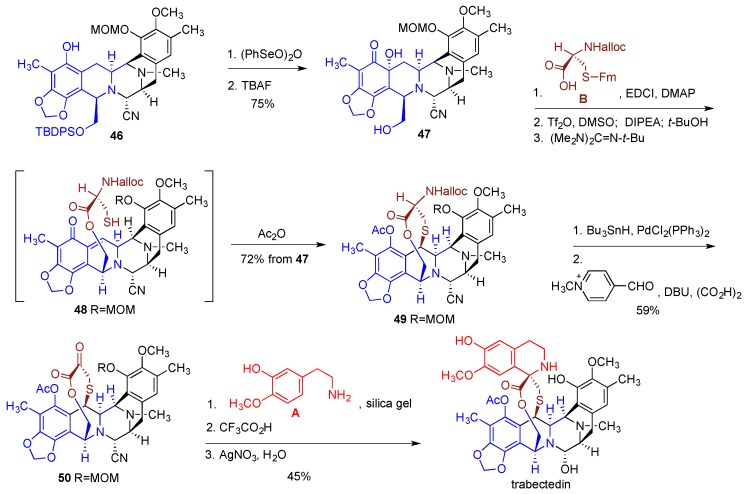
Incorporation of fragments **A** and **B** and completion of the synthesis of trabectedin.

To incorporate the sulfur containing bridge (building block **B**) the phenol functionality in intermediate **46** was oxidized with benzene-seleninic anhydride to the α-hydroxyketone moiety and the TBDPS ether was removed with TBAF to afford **47** (Figure 10). Intermediate **47** was esterified with protected cysteine derivative **B**. Treatment of the resulting ester with Tf_2_O and DMSO and then with the Hunig base gave an electrophilic *o*-quinone methide functionality, which underwent intramolecular coupling with the thiol group released after the removal of the fluorenylmethyl protection with the Barton’s base (see **48**). Acetylation of the phenolic hydroxyl then gave bridged intermediate **49** incorporating fragments **B**, **C** and **D**. Removal of the Alloc protection and Rappaport deamination produced ketone **50**, which was reacted with the tyramine derivative **A** under Pictet-Spengler condition to complete the construction of the multicyclic framework of trabectedin. Finally, removal of the MOM protection and conversion of the aminonitrile functionality to the carbinolamine with AgNO_3_ completed the synthesis. The sequence of reactions leading to trabectedin from intermediate **46** involved 11 steps and proceeded in 14% overall yield.

**Figure 11:**
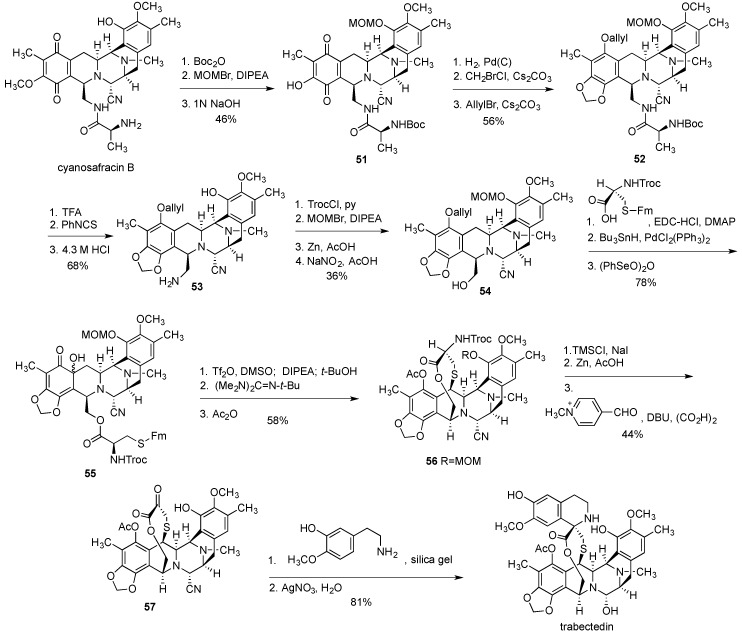
Semisynthesis of trabectedin from cyanosafracin B.

Overall, the synthesis involved 46 steps with 36 of them being part of the longest linear sequence. The overall yield for the longest linear sequence starting from sesamol **34** was 1%. This is quite respectable giving the complexity of the structure and the synthesis of trabectedin by Corey’s group undoubtedly represents an outstanding achievement. Yet, it provided only a temporary solution to the supply problem and more cost-effective synthetic processes were sought. In 2000, PharmaMar disclosed a semisynthetic route to trabectedin [197], which was eventually adopted and is used currently for the industrial preparation of multigram quantities of this cancer drug. The semisynthesis starts with cyanosafracin B [198] (Figure 11), an antibiotic of bacterial origin, obtained in kilogram quantities by fermentation of the bacteria *Pseudomonas fluorescens* [199].

The synthesis commenced by protecting the amino group and the phenolic oxygen with Boc and MOM functionalities, respectively, followed by the substitution of the methoxy moiety by the hydroxy group in the quinone ring to give **51** (Figure 11). The quinone ring was then reduced to hydroquinone and the resulting phenolic oxygens were converted to the methylenedioxy functionality and allyl ether to produce **52**. After the removal of MOM and Boc protecting groups, the liberated amine was subjected to Edman degradation to give amine **53**. Temporary protection of the primary amine as Troc carbamate, the introduction of the phenolic MOM, removal of Troc with Zn/AcOH was followed by the substitution of the amino group with the hydroxyl moiety using NaNO_2_/AcOH to result in **54**. The rest of the synthesis heavily relied on the transformations described in the above-discussed Corey’s sequences. Thus, the esterification of **54** with the protected cysteine and oxidation of the phenol to the α-hydroxy ketone in **55** was followed by the sequence of steps leading to the *o*-quinone methide formation and intramolecular addition of the thiol to give **56**. Finally, MOM removal and Rappaport deamination led to **57**, which was subjected to the Pictet-Spengler reaction and substitution of the cyano group by the hydroxyl to give trabectedin.

The total synthesis of trabectedin by Corey’s group and the subsequent semisynthetic approach by the PharmaMar scientists, currently utilized for the industrial preparation, serve as an excellent demonstration of the ability of synthesis to provide necessary quantities of a pharmacological agent for its clinical use. Indeed, multistep synthesis of complex natural products, such as those produced by marine invertebrates, should no longer be regarded as an academic exercise aimed to produce milligram quantities of the final product, but rather deserves to be viewed as a serious alternative capable of solving the supply problem no matter how complex the compound’s structure.

### 2.7. Synthetic Access to Marine Invertebrate Metabolites with Promising Anticancer Activities

Table 1 shows the examples of marine invertebrate metabolites selected on the basis of reported anticancer activities. The table includes metabolites, which we consider to be true promising anticancer agents as opposed to any marine invertebrate-derived natural products with reported cytotoxicity only. For such a selection we used any of the following criteria: (1) known modes of action; (2) demonstrated *in vivo* activity; or (3) having potential to be selective toward specific types of cancer. From the discussion in Section 2.5 and Section 2.6 it is clear that the number of steps in a synthesis is crucially important for the feasibility and practicality of material provision, as it determines the manpower, time and resource input, as well as the amount of waste and byproducts to be handled. Indeed, step count, or “step economy” as it is often referred to in the synthetic community [200,201], is key to the preparation of practical quantities of material. Because marine invertebrates are generally a poor source for metabolite provision, it is our hope that Table 1 will serve as guidance for researchers in the field as to what promising anticancer agents can be accessed via synthetic approaches.

We also hope that the table will be useful to synthetic chemists looking for a challenge presented by natural products with significant anticancer potential but poorly (long synthetic sequence) or currently inaccessible (N/A) through synthesis. From this perspective, we want to specifically draw attention to marine invertebrate metabolites with, in our opinion, most promising anticancer activities and currently poorly accessible as discussed in the following paragraphs.

**Figure 12:**
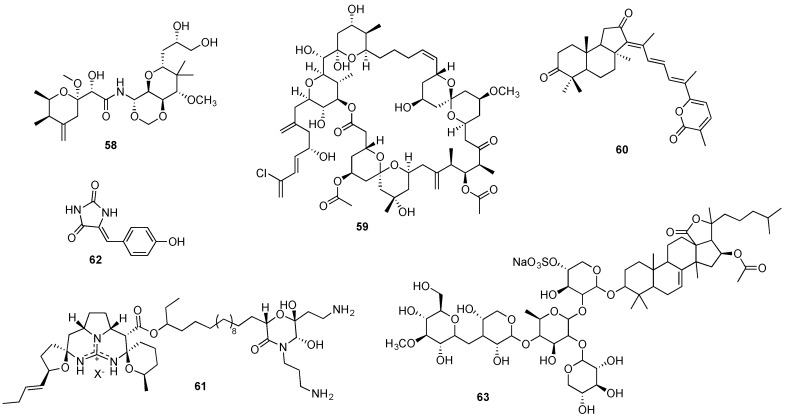
Structures of mycalamide A (**58**), spongistatin 1 (**59**), stelletin A (**60**), monanchocidin A (**61**), phenylmethylene hydantoin (**62**), and frondoside A (**63**).

Structurally unique, the pederin-type metabolite mycalamide A (**58**, Figure 12) initially isolated from an unspecified marine sponge *Mycale* sp. [279], received great attention since its discovery due to its *in vitro* cytotoxicity against several human cancer cell lines with nanomolar potency [280]. Mechanistic studies revealed that mycalamide A anticancer effect derives, at least partially, from its binding to the 80S ribosome and inhibition of protein synthesis [281]. Furthermore, the spongean metabolite was also reported to lead to the inhibition of oncogenic nuclear factors, which may indicate a potential cancer-preventive effect [280].

Mycalamide A has been reported from several sponge species, however being found in minute and inconsistent amounts ranging from 0.00025%–0.0011% of the sponge wet weight [279,282,283]. Despite this unattractive scenario, Page *et al.* [109] verified that through the in-sea aquaculture of the sponge *Mycale hentscheli*, mycalamide A could eventually be obtained in relatively higher amounts. Curiously, this metabolite was also obtained from the ascidian *Polysincraton* sp., but analogously to the previous reports, only 5.2 mg were obtained from 1000 g of the animal wet weight [284]. An alternative approach for the sustainable supply of mycalamide A through heterologous expression can also be hypothesized due to the structural similarity with pederin, for which the putative *trans*-AT-PKS cluster has been already identified [160].

This metabolite has been shown to be accessible through total synthesis and a number of such completed endeavors has been reported, of which the synthesis by Rawal and coworkers appears to be the shortest one, involving 33 steps [253]. It is thus unlikely that significant quantities of material can be prepared with the currently available synthetic sequences for its further preclinical evaluation. The presence of 10 stereocenters in the structure should be quite attractive to synthetic chemists seeking applications of their stereoselective synthetic methodologies. Given the promise of mycalamide A as an anticancer agent, the development of shorter syntheses is warranted.

Almost simultaneously reported by Pettit and Kobayashi groups, from the sponges *Spongia* sp. [285] and *Hyrtios altum* [286] respectively, spongistatin 1 (**59**, Figure 12) displayed an extremely potent and selective *in vivo* cytotoxic activity at picomolar concentrations in the NCI 60 panel of human cancer cell lines [287], including chemoresistant cancer cell lines [285,286]. Spongistatin 1 was found to retain its remarkable anticancer effect also *in vivo*, displaying a potent effect against several tumor xenografts without significant associated toxicity [288]. While spongistatin 1 anticancer mechanism has not yet been completely elucidated, its promising anticancer properties include an inhibitory action on tubulin polymerization [287], an anti-metastatic effect [288] and caspase-independent pro-apoptotic activity [289]. The spongean metabolite was isolated from five distinct sponges but in extremely low yields (0.003–0.17 mg/kg wet weight) [98], and the lack of further reports on its large-scale supply through aquaculture, indicates that such an approach may be unfeasible. Currently, the shortest synthesis of this metabolite was reported by the Smith’s group [274], which involves 27 steps. This synthesis is currently being optimized by this group [274] for a scalable gram-quantity production. The analysis of spongistatin’s 1 structure (**59**, Figure 12) reveals that such a goal presents a daunting challenge, but should be highly rewarding from both fundamental and applied science perspectives.

The spongean cytotoxic agents stelletin A (**60**, Figure 12) and monanchocidin A (**61**, Figure 12) hold great promise as potential candidates for further development as anticancer agents [290,291]. Both metabolites were found to induce autophagy in human cancer cell lines [292,293], in addition to a significant *in vitro* antiproliferative effect against leukemia cell lines [291,292,294]. Furthermore, monanchocidin A cytotoxic effect against genitourinary cancer cell lines was remarkably selective, compared to non-malignant genitourinary cells [293].

Although identified in several sponge species, stelletin A’s low concentration in these natural matrixes (<0.012% of sponge wet weight) clearly limits an eventual large-scale supply directly from the natural sources [290,295,296]. The isolation yield of monanchocidin’s A from *Monachora pulchra* constitutes only 1.54 mg of the compound from nearly 50 g of sponge dry weight [291,297]. No completed total syntheses for either metabolite have been reported, although structurally (**60** and **61** in Figure 12) they appear to represent ample opportunities for synthetic chemists to test new methodologies and ultimately develop scalable syntheses of these natural products.

Isolated from the marine sponge *Hemimycale arabica*, phenylmethylene hydantoin (**62**, Figure 12) and several derivatives exhibited a significant *in vitro* cytotoxic effect against human cancer cells as well as *in vivo* activity in PC-3M and MDA-MB-231 xenografts [298,299,300]. Remarkably, the spongean metabolite and some of its synthetic derivatives were also found to display anti-invasive and anti-metastatic properties, both *in vitro* and *in vivo* [299,300]. This compound has a very simple structure and is commercially available or easily accessible *via* one-step synthesis from commercially available inexpensive starting materials. This easy access to large quantities of phenylmethylene hydantoin and promising anticancer potential should encourage its further preclinical studies.

Targeting several signaling pathways, the triterpenoid glycoside frondoside A (**63**, Figure 12) has been reported as a promising anticancer agent leading to an *in vitro* and *in vivo* growth inhibitory effect in several human cancer cell lines [301,302]. Furthermore, frondoside A anticancer effect was also associated to its anti-invasive, anti-migratory and anti-metastatic properties, reported in breast [303,304] and lung cancer cells [305]. Frondoside A was initially reported more than 20 years ago as a metabolic product of the sea cucumber *Cucumaria frondosa* [306] and was later discovered in *Cucumaria okhotensis* in extremely low concentrations (4.4 mg from 1.4 kg of dried animal residue) [307]. So far no synthetic studies have been reported for this triterpene glycoside, although it appears that its terpenoid and glycosidic portions of the molecule should present an interesting challenge to the synthetic community to apply the ample current know-how for the construction of these structural frameworks.

### 2.8. Hybrid Strategies

In addition to the mainstream and widely used classical semisynthetic strategy, further elegant technologies combining microbial genetics, fermentation, and chemical synthesis can provide key intermediates or even the desired metabolite produced in minute quantities by a marine invertebrate. The identification of gene clusters involved in the production of secondary metabolites allows the generation of new intermediates through combinatorial biosynthesis, exploiting the ability of biological systems to generate complex chemistry, for example, *via* the manipulation of components from a metabolic pathway such as PKS and NRPS genes, or through the combination of genes from distinct organisms [308,309,310,311]. Additional approaches are mutasynthesis and chembiosynthesis, which have been successfully applied in the commercial synthesis of several drugs [310].

Another technology involves the combination of molecular engineering, microbial fermentation, and chemical synthesis, which has been achieved for the production of the tubulin-interactive agent discodermolide (**21**, Figure 4). The generation of key intermediates by genetically modified *Streptomyces* strains, carrying a mutated PKS gene and fed with specific unnatural precursors, facilitated the post-fermentation production of discodermolide by chemical synthesis [312]. Posteriorly to the remarkable achievement on the heterologous expression of patellamides, a novel cyclic peptide eptidemnamide was generated by modification of the patellamide genes [69]. The potential of these combined approaches relies not only in the possibility of generating intermediates of natural products obtained in insufficient quantities from their natural source, but also in the construction of libraries of analogues, structurally related to a lead compound, that may have improved pharmacological activity.

## 3. Future Prospects

The last two decades have witnessed intensive efforts to utilize marine invertebrates for the provision of molecules with anticancer activities. The importance of marine compounds in drug research is demonstrated by the fact that around 50% of the FDA-approved drugs during the period 1981–2014 are either marine metabolites or their synthetic analogues [313,314,315]. The US NCI estimates that more than 1% of marine natural products show antitumor properties as compared to the 0.01% of their terrestrial counterparts [316]. Gerwick and Moore [313] reported in 2012 that the success rate of discovery from the marine world for any type of clinical indication (seven drugs from 22,000 discovered molecular entities, *i.e.*, one drug per 3140 natural products described) is 1.7 to 3.3–fold higher than the industry average (one drug from 5000–10,000 tested compounds). However, the lack of a sustainable large-scale supply of marine-sourced drugs or drug candidates has been and continues to be one of the main challenges in pharmacology.

Wild harvest of marine invertebrates can in many cases provide sufficient amount of material for preclinical studies. However, clinical development and commercial production of successful agents necessitates large collections of marine organisms, which is environmentally unsustainable due to the poor abundance of their natural populations and low isolation yields of the bioactive metabolites. While the scale-up of marine invertebrate-derived metabolites for clinical development through aquaculture may play its part, this strategy can be only considered in a very limited number of cases. Even with the optimization of growth parameters this approach is usually economically daunting as described by the development of trabectedin. Despite the progress in culturing of invertebrate cells, this technique is far from being able to offer a sustainable supply of metabolites in the near future. In contrast, the recognition that a significant number of anticancer agents originally reported as invertebrate-derived may possibly have a bacterial origin, brings much hope to not only to solving the supply problem but also to the creation of new drug leads. Recombinant technologies such as the heterologous expression of biosynthetic pathways have been a major focus in recent years, and despite the above-mentioned limitations, the successful production of patellamides paves the way and demonstrates that this approach may be viable in the upcoming years.

Chemical synthesis has been and in all likelihood will continue to be indispensable in obtaining marine invertebrate-derived drugs and promising drug candidates in quantity. The fact that all four currently approved drugs are produced by synthesis is excellent testimony to the significance of this resource. As can be seen in the Figures contained in the current review, marine natural products stand out due to their complex structures incorporating multicyclic carbon skeletons and numerous stereogenic centers. On one hand, this raises the bar for organic chemists to develop scalable pathways to these formidable synthetic targets. On the other hand, this could be a blessing in disguise as these structures attract numerous organic chemists looking for an academic total synthesis journey and laying the paths to these complex molecules. These synthetic routes can often be later optimized for gram or even kilogram scale production, as we have seen in the cases of trabectedin and Halaven^®^. We hope that the synthetic chemistry described in this review as well as the discussion of the anticancer activities associated with many marine invertebrate-derived molecules will stimulate further interest in the synthetic community to help develop new life-saving medicines while advancing the science of chemical synthesis from the fundamental perspective.

## Figures and Tables

**Table 1:** Selected total or semisyntheses of marine invertebrate metabolites with reported promising anticancer activities.

Natural Product	Total or Semisynthesis			
	T/S ^a^	# of Steps	Starting Material	Reference
Aaptamine	T	5	6,7-dimethoxy-1-methylisoquinoline	[202]
4-Acetoxythorectidaeolide A	N/A	N/A	N/A	N/A
Adociaquinones A and B	T	3	2,5-dimethoxy-bicyclo[4.2.0]octa-1,3,5-triene	[203]
Agelastatin A	T	9	d-Aspartic acid	[204]
Agosterol A	T/S	23	Ergosterol	[205]
Aplidine (plitidepsin or dehydrodidemnin B)	T	3	d-Proline	[206]
Aplyronine A	T	15	(*R*)-3-(benzyloxy)-2 methylpropanal	[207]
Aplysiallene	T	16	(*S*,*S*)-Diepoxybutane	[208]
Arenastatin A (Cryptophycin-24)	T	14	(d)-*N*-Boc-tyrosine methyl ether	[209]
Austrasulfone	N/A	N/A	N/A	N/A
Avarol	T	11	Wieland-Miescher ketone	[210]
Avarone	T	10	Wieland-Miescher ketone	[210]
Bastadin 6	T	7	*N*-Boc-3,5-dibromotyramine	[211]
Bastadin 9	N/A	N/A	N/A	N/A
Bastadin 16	T	16	*p*-benzyloxybenzaldehyde	[212]
Batzellines A-D	T	12	4-aminoveratrol	[213]
Botryllamide A	N/A	N/A	N/A	N/A
Bryostatin-1	T	31	1,3-propanediol	[214]
Bryostatin-1	T	46	(*R*)-Isobutyl lactate	[215]
Candidaspongiolides A and B	N/A	N/A	N/A	N/A
Caulibugulone A	T	5	2,5-dihydroxybenzaldehyde	[216]
Cephalostatin 1	T	33	*trans*-androsterone	[217]
Chondropsin A	N/A	N/A	N/A	N/A
Clionamine B	S	11	Tigogenin	[218]
Comaparvin	N/A	N/A	NA	N/A
Cortistatin A	T	19	Prednisone	[219]
Crambescidin-816	N/A	N/A	N/A	N/A
Cytarabine	N/A ^b^	N/A	N/A	N/A
13-Deoxytedanolide	T	27	Methyl 3-oxopentanoate	[220]
Diacarnoxide B	N/A	N/A	N/A	N/A
Diazonamide A	T	20	7-benzyloxyindol	[221]
Dictyoceratin C	T	11	4-Hydroxy-3-methylbenzoic acid	[222]
Dictyostatin-1	T	19	2-Vinyl-1,3-dioxolane	[223]
Didemnin B	T	9	Ethyl lactate	[224]
Discodermolide	T	36	*trans*-Pentadiene	[225]
Dolastatin-10	T	11	(*S*)-Boc-proline	[226]
Dolastatin-15	T	10	l-Hydroxyisovalelic acid	[227]
Eleutherobin	T	27	(*R*)-(−)-α-Phellandrene	[228]
Ethylsmenoquinone	N/A	N/A	N/A	N/A
Fascaplysin	T	2	Tryptamine	[229]
Fijianolides A and B (Laulimalide)	N/A	N/A	N/A	N/A
Frondoside A	N/A	N/A	N/A	N/A
Furospinosulin-1	T	4	3-(3-furyl)propan-1-ol	[230]
Furospongolide	T	8	Geranyl acetate	[231]
Geodiamolide A	T	15	(−)-4-methylbutyrolactone	[232]
Geodiamolide B	T	13	d-tyrosine benzyl ester	[233]
Geodiamolide H	N/A	N/A	N/A	N/A
Girodazole	T	8		[234]
Halichondramide	N/A	N/A	N/A	N/A
Halichondrin B	T	38	2-deoxy-l-arabinose diethyl thioacetal 4,5-acetonide	[235]
Hemiasterlin A	N/A	N/A	N/A	N/A
Heteronemin	N/A	N/A	N/A	N/A
Hippuristanol	S	15	Hydrocortisone	[236]
14-Hydroxymethylxestoquinone	N/A	N/A	N/A	N/A
7-Hydroxyneolamellarin A	N/A	N/A	N/A	N/A
Z-4-Hydroxyphenylmethylene	CA ^d^	N/A	N/A	N/A
Hyrtioreticulins A and B	N/A	N/A	N/A	N/A
Ianthelline	T	5	3,5-dibromo-4-hydroxybenzaldehyde	[237]
Iejimalides B and C	T	15	Methyl (*E*)-3-bromo-2-methylacrylate	[238]
Ilimaquinone	T	17	Wieland-Miescher enone	[239]
Irciniastatin A (psymberin)	T	31	(−)-pantolactone	[240]
Irciniasulfonic acid	T	11	Hex-1-yne	[241]
Isobatzellines A-D	T	13	4-aminoveratrol	[213]
Kahalalide F	T	10	2-chlorotrityl chloride-resin	[242]
Kendarimide A	N/A	N/A	N/A	N/A
Lamellarin D	T	11	Vanillin and isovanillin	[243]
Lasonolide A	T	34	2,2,5-Trimethyl-1,3-dioxane-5-carbaldehyde	[244]
Latrunculin A	T	13	l-cysteine ethyl ester hydrochloride	[245]
Laulimalide	T	7	3,4-dihydro-2[2-methyl-4-pentyn-1-yl]-4-(phenylmethoxy)	[246]
Leucettamol A	N/A	N/A	N/A	N/A
Luffariellolide	T	8	Geranyl Bromide	[247]
Makaluvamines A, C	T	15	2-bromo-5-methoxy-benzenamine	[248]
Manadosterols A and B	N/A	N/A	N/A	N/A
Manzamine A	T	18	2-(bromomethyl)-2-ethenyl-1,3-dioxolane	[249]
Meridianin E	T	11	5-bromo-2-hydroxybenzaldehyde	[250]
Microsclerodermin A	N/A	N/A	*S*-citronellol	[251] ^c^
Mirabilin G	N/A	N/A	N/A	N/A
Monanchocidin A	N/A	N/A	3-azidopropanoic acid	[252] ^c^
Mycalamide A	T	33	Diethyl-*D*-tartrate	[253]
Mycothiazole	T	16	4,4-dimethyl-5-(phenylmethoxy)-2-penten-1-ol	[254]
Myriaporone 3	T	27	Methyl-(*S*)-(+)-3-hydroxy-2-methylpropionate	[255]
Myriaporone 4	T	27	Methyl-(*S*)-(+)-3-hydroxy-2-methylpropionate	[255]
Nakiterpiosin	T	23	3-bromo-2-methylbenzenecarboxylic acid	[256]
Neoamphimedine	T	10	2,5-Dimethoxy-3-nitrobenzoate	[257]
Neopetrosiamides A and B	T	4	Resin-bound linear peptide	[258]
Netamine M	N/A	N/A	N/A	N/A
Ningalin B	T	8	6-Bromoveratraldehyde	[259]
Onnamide A	T	5	5-iodopentadienoic acid	[260]
Pachastrissamine (jaspine B)	T	8	6-heptenal	[261]
Palau’amine	T	28	3-cyclohexene-1-carboxylic acid	[262]
Pateamine A	T	29	Dimethyl l-malate and *S*-methyl 3-hydroxy-2-methylpropionate	[263]
Peloruside A	T	22	3-Methyl-1-butyne	[264]
Petrosaspongiolide M	N/A	N/A	N/A	N/A
Philinopside A and E	N/A	N/A	N/A	N/A
PM050489	T	35	1,3-Propanediol	[265]
PM060184	T	33	1,3-Propanediol	[265]
Psammaplin A	T	5	3-bromo-4-hydroxybenzaldahyde	[266]
Psammaplysene A	T	5	4-Iodophenol	[267]
Renieramycin M	T	21	*N*-Trityl-l-serine methyl ester	[268]
Ritterazine B	N/A	N/A	N/A	N/A
Salicylihalamide A	T	16	ethylene glycol	[269]
Sarcodictyin A	T	25	(+)-Carvone	[270]
Sceptrin	T	25	l-Glutamic acid	[271]
Secobatzellines A and B	T	12	2,4,5-trimethoxy-benzaldehyde	[272]
Simplextone C	N/A	N/A	N/A	N/A
Sipholenol A	N/A	N/A	N/A	N/A
Sodwanone V	N/A	N/A	N/A	N/A
Spisulosine (ES-285)	T	9	(*S*)-Garner’s aldehyde.	[273]
Spongiacidin C	N/A	N/A	N/A	N/A
Spongistatin 1 (Altohyrtin A)	T	27	(*S*)-glycidol	[274]
Stelletin A	N/A	N/A	N/A	N/A
Strongylophorine 8 and 26	N/A	N/A	N/A	N/A
Subereamolline A	T	10	2-Hydroxy-4-methoxybenzaldehyde	[275]
Thorectidaeolide A	N/A	N/A	N/A	N/A
Trabectedin	T	28	1,3-propanodiol	[276]
Variolin B	T	8	4-chloro-2-methylthiopyrimidine	[277]
Vitilevuamide	N/A	N/A	N/A	N/A
Waixenicin A	N/A	N/A	N/A	N/A
Zampanolide	T	20	d-(−)-Aspartic acid	[278]

^a^: T = total synthesis, S = hemisynthesis; ^b^: N/A = synthesis not available; ^c^: only partial synthesis reported; ^d^: commercially available.

## Abbreviations

The following abbreviations are used in this manuscript:

Alloc	Allyloxycarbonyl
Bn	Benzyl
Boc	*tert*-Butyloxycarbonyl
BuLi	*n*-Butyllithium
Cbz	Carboxybenzyl
CIP	2-Chloro-1,3-dimethylimidazolidinium hexafluorophosphate
DIBAL	Diisobutylaluminium hydride
DMSO	Dimethyl sulfoxide
EMA	European Medicines Agency
FDA	Food and Drug Administration
HOAt	1-Hydroxy-7-azabenzotriazole
MOM	Methoxymethyl
NCI	US National Cancer Institute
NHK	Nozaki-Hiyama-Kishi reaction
NRPS	Non-ribosomal peptide synthetase
PKC	Protein kinase C
PLD	Pegylated liposomal doxoribucin
PPTS	Pyridinium *p*-toluenesulfonate
TBAF	Tetra-*n*-butylammonium fluoride
TBDPS	*tert*-Butyldiphenylsilyl
TBS	*tert*-Butyldimethylsilyl
Tf_2_O	Trifluoromethanesulfonic anhydride
THF	Tetrahydrofuran
*trans*-AT-PKS	*trans* Acyltransferase-polyketide synthase
Troc	Trichloroethyl chloroformate

## References

[1] Blunt J.W., Copp B.R., Keyzers R.A., Munro M.H., Prinsep M.R. (2016). Marine natural products. Nat. Prod. Rep..

[2] Newman D.J., Cragg G.M. (2004). Marine natural products and related compounds in clinical and advanced preclinical trials. J. Nat. Prod..

[3] Bailly C. (2009). Ready for a comeback of natural products in oncology. Biochem. Pharmacol..

[4] Kinghorn A.D., Chin Y.-W., Swanson S.M. (2009). Discovery of natural product anticancer agents from biodiverse organisms. Curr. Opin. Drug. Discov. Dev..

[5] Nobili S., Lippi D., Witort E., Donnini M., Bausi L., Mini E., Capaccioli S. (2009). Natural compounds for cancer treatment and prevention. Pharmacol. Res..

[6] Martins A., Vieira H., Gaspar H., Santos S. (2014). Marketed marine natural products in the pharmaceutical and cosmeceutical industries: Tips for success. Mar. Drugs.

[7] Newman D.J., Giddings L.-A. (2014). Natural products as leads to antitumor drugs. Phytochem. Rev..

[8] Simmons T.L., Andrianasolo E., McPhail K., Flatt P., Gerwick W.H. (2005). Marine natural products as anticancer drugs. Mol. Cancer. Ther..

[9] Schumacher M., Kelkel M., Dicato M., Diederich M. (2011). Gold from the sea: Marine compounds as inhibitors of the hallmarks of cancer. Biotechnol. Adv..

[10] Sawadogo W.R., Schumacher M., Teiten M.H., Cerella C., Dicato M., Diederich M. (2013). A survey of marine natural compounds and their derivatives with anti-cancer activity reported in 2011. Molecules.

[11] Stonik V.A., Fedorov S.N. (2014). Marine low molecular weight natural products as potential cancer preventive compounds. Mar. Drugs.

[12] Sawadogo W.R., Boly R., Cerella C., Teiten M.H., Dicato M., Diederich M. (2015). A Survey of marine natural compounds and their derivatives with anti-cancer activity reported in 2012. Molecules.

[13] Mayer A.M.S., Glaser K.B., Cuevas C., Jacobs R.S., Kem W., Little R.D., McIntosh J.M., Newman D.J., Potts B.C., Shuster D.E. (2010). The odyssey of marine pharmaceuticals: A current pipeline perspective. Trends Pharmacol. Sci..

[14] Newman D.J., Cragg G.M. (2014). Marine-sourced anti-cancer and cancer pain control agents in clinical and late preclinical development. Mar. Drugs.

[15] Hu Y., Chen J., Hu G., Yu J., Zhu X., Lin Y., Chen S., Yuan J. (2015). Statistical research on the bioactivity of new marine natural products discovered during 28 years from 1985 to 2012. Mar. Drugs.

[16] Mayer A.M., Gustafson K.R. (2004). Marine pharmacology in 2001–2002: Antitumour and cytotoxic compounds. Eur. J. Cancer.

[17] Mayer A.M., Gustafson K.R. (2006). Marine pharmacology in 2003–2004: Anti-tumour and cytotoxic compounds. Eur. J. Cancer.

[18] Mayer A.M., Gustafson K.R. (2008). Marine pharmacology in 2005–2006: Antitumour and cytotoxic compounds. Eur. J. Cancer.

[19] Williams D.H., Stone M.J., Hauck P.R., Rahman S.K. (1989). Why are secondary metabolites (natural products) biosynthesized?. J. Nat. Prod..

[20] Firn R.D., Jones C.G. (2003). Natural products: A simple model to explain chemical diversity. Nat. Prod. Rep..

[21] Paul V.J., Puglisi M.P. (2004). Chemical mediation of interactions among marine organisms. Nat. Prod. Rep..

[22] Paul V.J., Puglisi M.P., Ritson-Williams R. (2006). Marine chemical ecology. Nat. Prod. Rep..

[23] Paul V.J., Ritson-Williams R., Sharp K. (2011). Marine chemical ecology in benthic environments. Nat. Prod. Rep..

[24] Cooper E.L., Yao D. (2012). Diving for drugs: Tunicate anticancer compounds. Drug Discov. Today.

[25] Imperatore C., Aiello A., D’Aniello F., Senese M., Menna M. (2014). Alkaloids from marine invertebrates as important leads for anticancer drugs discovery and development. Molecules.

[26] Mehbub M.F., Lei J., Franco C., Zhang W. (2014). Marine sponge derived natural products between 2001 and 2010: Trends and opportunities for discovery of bioactives. Mar. Drugs.

[27] Pejin B., Mojovic M., Savic A.G. (2014). Novel and highly potent antitumour natural products from cnidarians of marine origin. Nat. Prod. Res..

[28] Bergmann W., Feeney R.J. (1950). The isolation of a new thymine pentoside from sponges 1. J. Am. Chem. Soc..

[29] Bergmann W., Feeney R.J. (1951). Contributions to the study of marine products. XXXII. The nucleosides of sponges I. J. Org. Chem..

[30] Rinehart K.L., Gloer J.B., Hughes R.G., Renis H.E., McGovren J.P., Swynenberg E.B., Stringfellow D.A., Kuentzel S.L., Li L.H. (1981). Didemnins: Antiviral and antitumor depsipeptides from a Caribbean tunicate. Science.

[31] Nuijen B., Bouma M., Manada C., Jimeno J.M., Schellens J.H.M., Bult A., Beijnen J.H. (2000). Pharmaceutical development of anticancer agents derived from marine sources. Anticancer Drugs.

[32] Leal M.C., Puga J., Serôdio J., Gomes N.C., Calado R. (2012). Trends in the discovery of new marine natural products from invertebrates over the last two decades—Where and what are we bioprospecting?. PLoS ONE.

[33] Molinski T.F., Dalisay D.S., Lievens S.L., Saludes J.P. (2009). Drug development from marine natural products. Nat. Rev. Drug Discov..

[34] Bhatnagar I., Kim S.-K. (2010). Marine antitumor drugs: Status, shortfalls and strategies. Mar. Drugs.

[35] Marine Pharmaceuticals The Clinical Pipeline. http://marinepharmacology.midwestern.edu/clinPipeline.htm.

[36] National Cancer Institute Cytarabine. http://www.cancer.gov/about-cancer/treatment/drugs/cytarabine.

[37] European Medicines Agency Cytarabine EU/3/11/942. http://www.ema.europa.eu/ema/index.jsp?curl=pages/medicines/human/orphans/2012/02/human_orphan_001014.jsp&mid=WC0b01ac058001d12b&source=homeMedSearch.

[38] National Cancer Institute Cytarabine Liposome. http://www.cancer.gov/about-cancer/treatment/drugs/cytarabineliposome.

[39] European Medicines Agency DepoCyte. http://www.ema.europa.eu/ema/index.jsp?curl=pages/medicines/human/medicines/000317/human_med_000740.jsp&mid=WC0b01ac058001d124.

[40] ClinicalTrials.gov Cytarabine (Open Studies). https://www.clinicaltrials.gov/ct2/results?term=cytarabine&recr=Open&no_unk=Y.

[41] EU Clinical Trials Register Clinical trials for Cytarabine. https://www.clinicaltrialsregister.eu/ctr-search/search?query=Cytarabine+&status=ongoing.

[42] European Medicines Agency Trabectedin. http://www.ema.europa.eu/ema/index.jsp?curl=pages/medicines/human/medicines/000773/human_med_001165.jsp&mid=WC0b01ac058001d124.

[43] National Cancer Institute Trabectedin. http://www.cancer.gov/about-cancer/treatment/drugs/trabectedin.

[44] Uemura D., Takahashi K., Yamamoto T., Katayama C., Tanaka J., Okumura Y., Hirata Y. (1985). Norhalichondrin A: An antitumor polyether macrolide from a marine sponge. J. Am. Chem. Soc..

[45] Hirata Y., Uemura D. (1986). Halichondrins—Antitumor polyether macrolides from a marine sponge. Pure Appl. Chem..

[46] European Medicines Agency Halaven. http://www.ema.europa.eu/ema/index.jsp?curl=pages/medicines/human/medicines/002084/human_med_001427.jsp&mid=WC0b01ac058001d124.

[47] National Cancer Institute Eribulin Mesylate. http://www.cancer.gov/about-cancer/treatment/drugs/eribulinmesylate.

[48] EU Clinical Trials Register Clinical trials for Eribulin Mesylate. https://www.clinicaltrialsregister.eu/ctr-search/search?query=Eribulin+Mesylate+&status=ongoing.

[49] ClinicalTrials.gov Eribulin Mesylate (ongoing). https://www.clinicaltrials.gov/ct2/results?term=Eribulin+Mesylate&recr=Open&no_unk=Y.

[50] National Cancer Institute Brentuximab Vedotin. http://www.cancer.gov/about-cancer/treatment/drugs/brentuximabvedotin.

[51] European Medicines Agency Adcetris. http://www.ema.europa.eu/ema/index.jsp?curl=pages/medicines/human/medicines/002455/human_med_001588.jsp&mid=WC0b01ac058001d124.

[52] Pettit G.R., Kamano Y., Herald C.L., Tuinman A.A., Boettner F.E., Kizu H., Schmidt J.M., Baczynskyj L., Tomer K.B., Bontems R.J. (1987). The isolation and structure of a remarkable marine animal antineoplastic constituent: Dolastatin 10. J. Am. Chem. Soc..

[53] Vaishampayan U., Glode M., Du W., Kraft A., Hudes G., Wright J., Hussain M. (2000). Phase II study of dolastatin 10 in patients with hormone-refractory metastatic prostate adenocarcinoma. Clin. Cancer Res..

[54] Hoffman M., Blessing J., Lentz S. (2003). A phase II trial of dolastatin-10 in recurrent platinum-sensitive ovarian carcinoma: A gynecologic oncology group study. Gynecol. Oncol..

[55] Thoms C., Schupp P. (2005). Biotechnological potential of marine sponges and their associated bacteria as producers of new pharmaceuticals (Part II). JIBL.

[56] Schmitz F.J., Bowden B.F., Toth S.I., Attaway D.H., Zaborsky O.R. (1993). Antitumor and cytotoxic compounds from marine organisms. Marine Biotechnolog. Pharmaceutical and Bioactive Natural Products.

[57] Allen M.J., Jaspars M. (2009). Realizing the potential of marine biotechnology: Challenges & opportunities. Ind. Biotechnol..

[58] Desbois A.P. (2014). How might we increase success in marine-based drug discovery?. Expert Opin. Drug Discov..

[59] Crawford J.M., Clardy J. (2011). Bacterial symbionts and natural products. Chem. Commun..

[60] Hochmuth T., Niederkrüger H., Gernert C., Siegl A., Taudien S., Platzer M., Crews P., Hentschel U., Piel J. (2010). Linking chemical and microbial diversity in marine sponges: Possible role for poribacteria as producers of methyl-branched fatty acids. ChemBioChem.

[61] Webster N.S., Taylor M.W., Benham F., Lücker S., Rattei T., Whalan S., Horn M., Wagner M. (2010). Deep sequencing reveals exceptional diversity and modes of transmission for bacterial sponge symbionts. Environ. Microbiol..

[62] Hentschel U., Hopke J., Horn M., Friedrich A.B., Wagner M., Hacker J., Moore B.S. (2002). Molecular evidence for a uniform microbial community in sponges from different oceans. Appl. Environ. Microbiol..

[63] Schmitt S., Wehrl M., Bayer K., Siegl A., Hentschel U. (2007). Marine sponges as models for commensal microbe-host interactions. Symbiosis.

[64] Thacker W., Starnes S. (2003). Host specificity of the symbiotic cyanobacterium *Oscillatoria spongeliae* in marine sponges, *Dysidea* spp.. Mar. Biol..

[65] Usher K.M., Fromont J., Sutton D.C., Toze S. (2004). The biogeography and phylogeny of unicellular cyanobacterial symbionts in sponges from Australia and the Mediterranean. Microb. Ecol..

[66] Santos-Gandelman J.F., Giambiagi-deMarval M., Oelemann W.M.R., Laport M.S. (2014). Biotechnological potential of sponge-associated bacteria. Curr. Pharm. Biotechnol..

[67] Taylor M.W., Radax R., Steger D., Wagner M. (2007). Sponge-associated microorganisms: Evolution, ecology, and biotechnological potential. Microbiol. Mol. Biol. Rev..

[68] Thomas T.R.A., Kavlekar D.P., LokaBharathi P.A. (2010). Marine drugs from sponge-microbe association—A review. Mar. Drugs.

[69] Donia M.S., Hathaway B.J., Sudek S., Haygood M.G., Rosovitz M.J., Ravel J., Schmidt E.W. (2006). Natural combinatorial peptide libraries in cyanobacterial symbionts of marine ascidians. Nat. Chem. Biol..

[70] Donia M.S., Fricke W.F., Ravel J., Schmidt E.W. (2011). Variation in tropical reef symbiont metagenomes defined by secondary metabolism. PLoS ONE.

[71] Schmidt E.W., Donia M.S., McIntosh J.A., Fricke W.F., Ravel J. (2012). Origin and variation of tunicate secondary metabolites. J. Nat. Prod..

[72] Moore B.S. (1999). Biosynthesis of marine natural products: Microorganisms and macroalgae. Nat. Prod. Rep..

[73] Moore B.S. (2006). Biosynthesis of marine natural products: Macroorganisms (Part B). Nat. Prod. Rep..

[74] Schmidt E.W., Donia M.S. (2010). Life in cellulose houses: Symbiotic bacterial biosynthesis of ascidian drugs and drug leads. Curr. Opin. Biotechnol..

[75] Waters A.L., Hill R.T., Place A.R., Hamann M.T. (2010). The expanding role of marine microbes in pharmaceutical development. Curr. Opin. Biotechnol..

[76] Gomes N.G.M., Lefranc F., Kijjoa A., Kiss R. (2015). Can some marine-derived fungal metabolites become actual anticancer agents?. Mar. Drugs.

[77] König G.M., Kehraus S., Seibert S.F., Abdel-Lateff A., Müller D. (2006). Natural products from marine organisms and their associated microbes. ChemBioChem.

[78] Uria A., Piel J. (2009). Cultivation-independent approaches to investigate the chemistry of marine symbiotic bacteria. Phytochem. Rev..

[79] Giddings L.-A., Newman D.J. (2013). Microbial natural products: Molecular blueprints for antitumor drugs. J. Ind. Microbiol. Biotechnol..

[80] Tsukimoto M., Nagaoka M., Shishido Y., Fujimoto J., Nishisaka F., Matsumoto S., Harunari E., Imada C., Matsuzaki T. (2011). Bacterial production of the tunicate-derived antitumor cyclic depsipeptide didemnin B. J. Nat. Prod..

[81] Harvey B.M., Mironenko T., Sun Y., Hong H., Deng Z., Leadlay P.F., Weissman K.J., Haydock S.F. (2007). Insights into polyether biosynthesis from analysis of the nigericin biosynthetic gene cluster in *Streptomyces* sp. DSM4137. Chem. Biol..

[82] Demydchuk Y., Sun Y., Hong H., Staunton J., Spencer J.B., Leadlay P.F. (2008). Analysis of the tetronomycin gene cluster: Insights into the biosynthesis of a polyether tetronate antibiotic. ChemBioChem.

[83] Harrigan G.G., Yoshida W.Y., Moore R.E., Nagle D.G., Park P.U., Biggs J., Paul V.J., Mooberry S.L., Corbett T.H., Valeriote F.A. (1998). Isolation, structure determination, and biological activity of dolastatin 12 and lyngbyastatin 1 from *Lyngbya majuscula*/*Schizothrix calcicola* cyanobacterial assemblages. J. Nat. Prod..

[84] Luesch H., Moore R.E., Paul V.J., Mooberry S.L., Corbett T.H. (2001). Isolation of dolastatin 10 from the marine cyanobacterium *Symploca* species VP642 and total stereochemistry and biological evaluation of its analogue symplostatin 1. J. Nat. Prod..

[85] Barr P.M., Lazarus H.M., Cooper B.W., Schluchter M.D., Panneerselvam A., Jacobberger J.W., Hsu J.W., Janakiraman N., Simic A., Dowlati A. (2009). Phase II study of bryostatin 1 and vincristine for aggressive non-Hodgkin lymphoma relapsing after an autologous stem cell transplant. Am. J. Hematol..

[86] Morgan R.J., Leong L., Chow W., Gandara D., Frankel P., Garcia A., Lenz H.-J., Doroshow J.H. (2012). Phase II trial of bryostatin-1 in combination with cisplatin in patients with recurrent or persistent epithelial ovarian cancer: A California cancer consortium study. Investig. New Drugs.

[87] Davidson S.K., Allen S.W., Lim G.E., Anderson C.M., Haygood M.G. (2001). Evidence for the biosynthesis of bryostatins by the bacterial symbiont, *Candidatus Endobugula sertula*, of the bryozoan *Bugula neritina*. Appl. Environ. Microbiol..

[88] Sudek S., Lopanik N.B., Waggoner L.E., Hildebrand M., Anderson C., Liu H., Patel A., Sherman D.H., Haygood M.G. (2007). Identification of the putative bryostatin polyketide synthase gene cluster from “*Candidatus Endobugula sertula*”, the uncultivated microbial symbiont of the marine bryozoans *Bugula neritina*. J. Nat. Prod..

[89] Lopanik N.B., Lindquist N., Targett N. (2004). Potent cytotoxins produced by a microbial symbiont protect host larvae from predation. Oecologia.

[90] Hamann M.T., Scheuer P.J. (1993). Kahalalide F: A bioactive depsipeptide from the sacoglossan mollusk *Elysia rufescens* and the green alga *Bryopsis* sp.. J. Am. Chem. Soc..

[91] Hamann M.T., Otto C.S., Scheuer P.J., Dunbar D.C. (1996). Kahalalides: Bioactive peptides from a marine mollusk *Elysia rufescens* and its algal diet *Bryopsis* sp.. J. Org. Chem..

[92] Enticknap J., Hamann M.T., Hill R.T., Rao K.V. (2005). Kahalalide-Producing Bacteria.

[93] Long B.H., Carboni J.M., Wasserman A.J., Cornell L.A., Casazza A.M., Jensen P.R., Lindel T., Fenical W., Fairchild C.R. (1998). Eleutherobin, a novel cytotoxic agent that induces tubulin polymerization, is similar to paclitaxel (Taxol). Cancer Res..

[94] Cinel B., Roberge M., Behrisch H., Van Ofwegen L., Castro C.B., Andersen R.J. (2000). Antimitotic diterpenes from *Erythropodium caribaeorum* test pharmacophore models for microtubule stabilization. Org. Lett..

[95] Piel J. (2004). Metabolites from symbiotic bacteria. Nat. Prod. Rep..

[96] Schmidt E.W. (2008). Trading molecules and tracking targets in symbiotic interactions. Nat. Chem. Biol..

[97] Piel J. (2009). Metabolites from symbiotic bacteria. Nat. Prod. Rep..

[98] Radjasa O.K., Vaske Y.M., Navarro G., Vervoort H.C., Tenney K., Linington R.G., Crews P. (2011). Highlights of marine invertebrate-derived biosynthetic products: Their biomedical potential and possible production by microbial associants. Bioorg. Med. Chem..

[99] Garson M.J., Simpson J.S. (2004). Marine isocyanides and related natural products–structure, biosynthesis and ecology. Nat. Prod. Rep..

[100] Gulder T.A.M., Moore B.S. (2009). Chasing the treasures of the sea—bacterial marine natural products. Curr. Opin. Microbiol..

[101] Piel J. (2006). Bacterial symbionts: Prospects for the sustainable production of invertebrate-derived pharmaceuticals. Curr. Med. Chem..

[102] Simmons T.L., Coates R.C., Clark B.R., Engene N., Gonzalez D., Esquenazi E., Dorrestein P.C., Gerwick W.H. (2008). Biosynthetic origin of natural products isolated from marine microorganism-invertebrate assemblages. Proc. Natl. Acad. Sci. USA.

[103] Hildebrand M., Waggoner L.E., Lim G.E., Sharp K.H., Ridley C.P., Haygood M.G. (2004). Approaches to identifify, clone, and express symbiont bioactive metabolite genes. Nat. Prod. Rep..

[104] Pomponi S.A. (1999). The bioprocess—Technological potential of the sea. J. Biotechnol..

[105] Munro M.H.G., Blunt J.W., Dumdei E.J., Hickford S.J.H., Lill R.E., Li S.X., Battershill C.N., Duckworth A.R. (1999). The discovery and development of marine compounds with pharmaceutical potential. J. Biotechnol..

[106] Schaufelberger D.E., Koleck M.P., Beutler J.A., Vatakis A.M., Alvarado A.B., Andrews P., Marzo L.V., Muschik G.M., Roach J., Ross J.T. (1991). The large-scale isolation of bryostatin 1 from *Bugula neritina* following good manufacturing practices. J. Nat. Prod..

[107] Duckworth A., Battershill C.N. (2003). Sponge aquaculture for the production of biologically active metabolites: The influence of farming protocols and environment. Aquaculture.

[108] Van Treeck P., Eisinger M., Müller J., Paster M., Schuhmacher H. (2003). Mariculture trials with Mediterranean sponge species: The exploitation of an old natural resource with sustainable and novel methods. Aquaculture.

[109] Page M.J., Northcote P.T., Webb V.L., Mackey S., Handley S.J. (2005). Aquaculture trials for the production of biologically active metabolites in the New Zealand sponge *Mycale hentscheli* (Demospongiae: Poecilosclerida). Aquaculture.

[110] Handley S.J., Page M.J., Northcote P.T. (2006). Anti-cancer sponge: The race is on for aquaculture supply. Water Atmos..

[111] Taglialatela-Scafati O., Deo-Jangra U., Campbell M., Roberge M., Andersen R.J. (2002). Diterpenoids from cultured Erythropodium caribaeorum. Org. Lett..

[112] Osinga R., Tramper J., Wijffels R.H. (1998). Cultivation of marine sponges for metabolite production: Applications for biotechnology?. Trends Biotechnol..

[113] Belarbi E.H., Gómez A.C., Chisti Y., Camacho F.C., Grima E.M. (2003). Producing drugs from marine sponges. Biotechnol. Adv..

[114] Duckworth A. (2009). Farming sponges to supply bioactive metabolites and bath sponges: A review. Mar. Biotechnol..

[115] Thornton R.S., Kerr R.G. (2002). Induction of pseudopterosin biosynthesis in the gorgonian *Pseudopterogorgia elisabethea*. J. Chem. Ecol..

[116] Belarbi E.H., Dominguez M.R., Garcia M.C.C., Gómez A.C., Camacho F.G., Grima E.M. (2003). Cultivation of explants of the marine sponge *Crambe crambe* in closed systems. Biomol. Eng..

[117] De Caralt S., Agell G., Uriz M.J. (2003). Long-term culture of sponge explants: Conditions enhancing survival and growth, and assessment of bioactivity. Biomol. Eng..

[118] Duckworth A.R., Samples G.A., Wright A.E., Pomponi S.A. (2003). *In vitro* culture of the tropical sponge *Axinella corrugata* (Demospongia): Effect of food cell concentration on growth, clearance rate and biosynthesis of stevensine. Mar. Biotechnol..

[119] Mendola D. (2003). Aquaculture of three phyla of marine invertebrates to yield bioactive metabolites: Process developments and economics. Biomol. Eng..

[120] Osinga R., de Beukelaer P.B., Meijer E.M., Tramper J., Wijffels R.H. (1999). Growth of the sponge *Pseudosuberites* (aff.) *andrewsi* in a closed system. J. Biotechnol..

[121] Koopmans M., Martens D., Wijffels R.H. (2009). Towards commercial production of sponge medicines. Mar. Drugs.

[122] Müller W.E.G., Zahn R.K., Gasic M.J., Dogovic N., Maidhof A., Becker C., Diehl-Seifert B., Eich E. (1985). Avarol, a cytostatically active compound from the marine sponge *Dysidea avara*. Comp. Biochem. Physiol. C.

[123] Sipkema D., Osinga R., Schatton W., Mendola D., Tramper J., Wijffels R.H. (2005). Large-scale production of pharmaceuticals by marine sponges: Sea, cell, or synthesis. Biotechnol. Bioeng..

[124] Müller W.E.G., Grebenjuk V.A., Le Pennec G., Schröder H.-C., Brümmer F., Hentschel U., Müller I.M., Breter H.-J. (2004). Sustainable production of bioactive compounds by sponges—Cell culture and gene cluster approach: A review. Mar. Biotechnol..

[125] De Caralt S., Uriz M.J., Wijffels R.H. (2008). Cell culture from sponges: Pluripotency and immortality. Trends Biotechnol..

[126] Sipkema D., van Wielink R., van Lammeren A.A.M., Tramper J., Osinga R., Wijffels R.H. (2003). Primmorphs from seven marine sponges: Formation and structure. J. Biotechnol..

[127] Sipkema D., Heilig H.G.H., Akkermans A.D.L., Osinga R., Tramper J., Wijffels R.H. (2003). Sponge cell culture? A molecular identification method for sponge cells. Mar. Biotechnol..

[128] Müller W.E.G., Krasko A., Le Pennec G., Steffen R., Wiens M., Ammar M.S.A., Müller I.M., Schröder H.-C. (2003). Molecular mechanism of spicule formation in the demosponge *Suberites domuncula*—Silicatein-myotrophin-collagen. Silicon Biominer..

[129] Müller W.E.G., Böhm M., Batel R., De Rosa S., Tommonaro G., Müller I.M., Schröder H.C. (2000). Application of cell culture for the production of bioactive compounds from sponges: Synthesis of avarol by primmorphs from *Dysidea avara*. J. Nat. Proc..

[130] Penesyan A., Kjelleberg S., Egan S. (2010). Development of novel drugs from marine surface associated microorganisms. Mar. Drugs.

[131] Zhang L., An R., Wang J., Sun N., Zhang S., Hu J., Kuai J. (2005). Exploring novel bioactive compounds from marine microbes. Curr. Opin. Microbiol..

[132] Proksch P., Putz A., Ortlepp S., Kjer J., Bayer M. (2010). Bioactive natural products from marine sponges and fungal endophytes. Phytochem. Rev..

[133] Rateb M.E., Ebel R. (2011). Secondary metabolites of fungi from marine habitats. Nat. Prod. Rep..

[134] Gerwick W.H., Fenner A.M. (2013). Drug discovery from marine microbes. Microb. Ecol..

[135] Xiong Z.Q., Wang J.F., Hao Y.Y., Wang Y. (2013). Recent advances in the discovery and development of marine microbial natural products. Mar. Drugs.

[136] Ward D.M., Weller R., Bateson M.M. (1990). 16S rRNA sequences reveal numerous uncultured microorganisms in a natural community. Nature.

[137] Pace N.R. (1997). A molecular view of microbial diversity and the biosphere. Science.

[138] Osinga R., Armstrong E., Burgess J.G., Hoffmann F., Reitner J., Schumann-Kindel G. (2001). Sponge-microbe associations and their importance for sponge bioprocess engineering. Hydrobiologia.

[139] Kaeberlein T., Lewis K., Epstein S.S. (2002). Isolating “uncultivable” microorganisms in pure culture in a simulated natural environment. Science.

[140] Barberel S.I., Walker J.R.L. (2000). The effect of aeration upon the secondary metabolism of microorganisms. Biotechnol. Genet. Eng. Rev..

[141] Pfefferle C., Theobald U., Gurtler H., Fiedler H. (2001). Improved secondary metabolite production in the genus *Streptosporangium* by optimization of the fermentation conditions. J. Biotechnol..

[142] Yan L., Boyd K.G., Burgess J.G. (2002). Surface attachment induced production of antimicrobial compounds by marine epiphytic bacteria using modified roller bottle cultivation. Mar. Biotechnol..

[143] Marman A., Aly A.H., Lin W., Wang B., Proksch P. (2014). Co-cultivation—A powerful emerging tool for enhancing the chemical diversity of microorganisms. Mar. Drugs.

[144] Hill R., Peraud O., Hamann M., Kasanah N. (2005). Manzamine-Producing Actinomycetes. U.S. Patent.

[145] Dunlap W.C., Battershill C.N., Liptrot C.H., Cobb R.E., Bourne D.G., Jaspars M., Long P.F., Newman D.J. (2007). Biomedicinals from the photosymbionts of marine invertebrates: A molecular approach. Methods.

[146] Salomon C.E., Magarvey N.A., Sherman D.H. (2004). Merging the potential of microbial genetics with biological and chemical diversity: An even brighter future for marine natural product drug discovery. Nat. Prod. Rep..

[147] Langer M., Gabor E.M., Liebeton K., Meurer G., Niehaus F., Schulze R., Eck J., Lorenz P. (2006). Metagenomics: An inexhaustible access to nature’s diversity. Biotechnol. J..

[148] Wilson M.C., Piel J. (2013). Metagenomic approaches for exploiting uncultivated bacteria as a resource for novel biosynthetic enzymology. Chem. Biol..

[149] Handelsman J. (2004). Metagenomics: Application of genomics to uncultured microorganisms. Microbiol. Mol. Biol. Rev..

[150] Fortman J.L., Sherman D.H. (2005). Utilizing the power of microbial genetics to bridge the gap between the promise and the application of marine natural products. ChemBioChem.

[151] Schofield M.M., Sherman D.H. (2013). Meta-omic characterization of prokaryotic gene clusters for natural product biosynthesis. Curr. Opin. Biotechnol..

[152] Handelsman J. (2005). Sorting out metagenomes. Nat. Biotechnol..

[153] Pfeifer B.A., Khosla C. (2001). Biosynthesis of polyketides in heterologous hosts. Microbiol. Mol. Biol. Rev..

[154] Pfeifer B.A., Admiraal S.J., Gramajo H., Cane D.E., Khosla C. (2001). Biosynthesis of complex polyketides in a metabolically engineered strain of *E. coli*. Science.

[155] Khosla C., Keasling J.D. (2003). Metabolic engineering for drug discovery and development. Nat. Rev. Drug Discov..

[156] Wenzel S.C., Gross F., Zhang Y., Fu J., Stewart A.F., Muller R. (2005). Heterologous expression of a myxobacterial natural products assembly line in pseudomonads via red/ET recombineering. Chem. Biol..

[157] Sarovich D.S., Pemberton J.M. (2007). pPSX: A novel vector for the cloning and heterologous expression of antitumor antibiotic gene clusters. Plasmid.

[158] Butzin N.C., Owen H.A., Collins M.L. (2010). A new system for heterologous expression of membrane proteins: *Rhodospirillum rubrum*. Protein Expr. Purif..

[159] Piel J. (2002). A polyketide synthase-peptide synthetase gene cluster from an uncultured bacterial symbiont of *Paederus beetles*. Proc. Natl. Acad. Sci. USA.

[160] Piel J., Butzke D., Fusetani N., Hui D., Platzer M., Wen G., Matsunaga S. (2005). Exploring the chemistry of uncultivated bacterial symbionts: Antitumor polyketides of the Pederin family. J. Nat. Prod..

[161] Xu Y., Kersten R.D., Nam S.-J., Lu L., Al-Suwailem A.M., Zheng H., Fenical W., Dorrestein P.C., Moore B.S., Qian P.-Y. (2012). Bacterial biosynthesis and maturation of the didemnin anti-cancer agents. J. Am. Chem. Soc..

[162] Long P.F., Dunlap W.C., Battershill C.N., Jaspars M. (2005). Shotgun cloning and heterologous expression of the patellamide gene cluster as a strategy to achieving sustained metabolite production. ChemBioChem.

[163] Schmidt E.W., Nelson J.T., Rasko D.A., Sudek S., Eisen J.A., Haygood M.G. (2005). Patellamide A and C biosynthesis by a microcin-like pathway in *Prochloron didemni*, the cyanobacterial symbiont of *Lissoclinum patella*. Proc. Natl. Acad. Sci. USA.

[164] Kennedy J., Marchesi J.R., Dobson A.D.W. (2007). Metagenomic approaches to exploit the biotechnological potential of the microbial consortia of marine sponges. Appl. Microbiol. Biotechnol..

[165] Velásquez J.E., van der Donk W.A. (2011). Genome mining for ribosomally synthesized natural products. Curr. Opin. Chem. Biol..

[166] Gunasekera S.P., Gunasekera M., Longley R.E., Schulte G.K. (1990). Discodermolide: A new bioactive polyhydroxylated lactone from the marine sponge Discodermia dissoluta. J. Org. Chem..

[167] Florence G.J., Gardner N.M., Paterson I. (2008). Development of practical syntheses of the marine anticancer agents discodermolide and dictyostatin. Nat. Prod. Rep..

[168] Smith A.B., Freeze B.S. (2007). (+)-Discodermolide: Total Synthesis, Construction of Novel Analogues, and Biological Evaluation. Tetrahedron.

[169] Mickel S.J., Sedelmeier G.H., Niederer D., Schuerch F., Grimler D., Koch G., Daeffler R., Osmani A., Hirni A., Schaer K. (2004). Large-Scale Synthesis of the Anti-Cancer Marine Natural Product (+)-Discodermolide. Part 2: Synthesis of Fragments C_1–6_ and C_9–14_. Org. Proc. Res. Dev..

[170] Mickel S.J., Sedelmeier G.H., Niederer D., Schuerch F., Koch G., Kuesters E., Daeffler R., Osmani A., Seeger-Weibel M., Schmid E., Hirni A. (2004). Large-Scale Synthesis of the Anti-Cancer Marine Natural Product (+)-Discodermolide. Part 3: Synthesis of Fragment C_15–21_. Org. Proc. Res. Dev..

[171] Mickel S.J., Sedelmeier G.H., Niederer D., Schuerch F., Seger M., Schreiner K., Daeffler R., Osmani A., Bixel D., Loiseleur O. (2004). Large-Scale Synthesis of the Anti-Cancer Marine Natural Product (+)-Discodermolide. Part 4: Preparation of Fragment C7–24. Org. Proc. Res. Dev..

[172] Mickel S.J., Niederer D., Daeffler R., Osmani A., Kuesters E., Schmid E., Schaer K., Gamboni R. (2004). Large-Scale Synthesis of the Anti-Cancer Marine Natural Product (+)-Discodermolide. Part 5: Linkage of Fragments C_1–6_ and C_7–24_ and Finale. Org. Proc. Res. Dev..

[173] Jackson K.L., Henderson J.A., Phillips A.J. (2009). The Halichondrins and E7389. Chem. Rev..

[174] Rinehart K.L., Holt T.G., Fregeau N.L., Stroh J.G., Keifer P.A., Sun F., Li L.H., Martin D.G. (1990). Ecteinascidins 729, 743, 745, 759A, 759B, and 770: Potent antitumor agents from the Caribbean tunicate *Ecteinascidia turbinate*. J. Org. Chem..

[175] Rinehart K.L., Holt T.G., Fregeau N.L., Stroh J.G., Keifer P.A., Sun F., Li L.H., Martin D.G. (1991). Ecteinascidins 729, 743, 745, 759A, 759B, and 770: Potent antitumor agents from the Caribbean tunicate *Ecteinascidia turbinate* [Erratum to document cited in CA113(9):75189d]. J. Org. Chem..

[176] Sakai R., Rinehart K.L., Guan Y., Wang A.H. (1992). Additional antitumor ecteinascidins from a Caribbean tunicate: Crystal structures and activities *in vivo*. Proc. Natl. Acad. Sci. USA.

[177] Guan Y., Sakai R., Rinehart K.L., Wang A.H. (1993). Molecular and crystal structures of ecteinascidins: Potent antitumor compounds from the Caribbean tunicate *Ecteinascidia turbinate*. J. Biomol. Struct. Dyn..

[178] Izbicka E., Lawrence R., Raymond E., Eckhardt G., Faircloth G., Jimeno J., Clark G., Von Hoff D.D. (1998). *In vitro* antitumor activity of the novel marine agent, Ecteinascidin-743 (ET-743, NSC-648766) against human tumors explanted from patients. Ann. Oncol..

[179] Hendriks H.R., Fiebig H.H., Giavazzi R., Langdon S.P., Jimeno J.M., Faircloth G.T. (1999). High antitumor activity of ET743 against human tumor xenografts from melanoma, non-small-cell lung and ovarian cancer. Ann. Oncol..

[180] Taamma A., Misset J.L., Riofrio M., Guzman C., Brain E., Lopez-Lazaro L., Rosing H., Jimeno J.M., Cvitkovic E. (2001). Phase I and Pharmacokinetic Study of Ecteinascidin-743, a New Marine Compound, Administered as a 24-hour Continuous Infusion in Patients With Solid Tumors. J. Clin. Oncol..

[181] Villalona-Calero M.A., Eckhardt S.G., Weiss G., Hidalgo M., Beijnen J.H., Van Kesteren C., Rosing H., Campbell E., Lopez-Lazaro L., Guzman C. (2002). A Phase I and Pharmacokinetic Study of Ecteinascidin-743 on Daily × 5 Schedule in Patients with Solid Malignancies. Clin. Cancer. Res..

[182] D’Incalci M., Jimeno J. (2003). Preclinical and clinical results with the natural marine product ET-743. Expert. Opin. Investig. Drugs.

[183] Janssen Products L.P. Yondelis (trabectedin). http://www.yondelis.com.

[184] Monk B.J., Herzog T.J., Kaye S.B., Krasner C.N., Vermorken J.B., Muggia F.M., Pujade-Lauraine E., Lisyanskaya A.S., Makhson A.N., Rolski J. (2010). Trabectedin Plus Pegylated Liposomal Doxorubicin in Recurrent Ovarian Cancer. J. Clin. Oncol..

[185] Krasner C.N., Poveda A., Herzog T.J., Vermorken J.B., Kaye S.B., Nieto A., Claret P.L., Park Y.C., Parekh T., Monk B.J. (2012). Patient-reported outcomes in relapsed ovarian cancer: Results from a randomized Phase III study of trabectedin with pegylated liposomal doxorubicin (PLD) *versus* PLD Alone. Gynecol. Oncol..

[186] Lebedinsky C., Gomez J., Park Y.C., Nieto A., Soto-Matos A., Parekh T., Alfaro V., Roy E., Lardelli P., Kahatt C. (2011). Trabectedin has a low cardiac risk profile: A comprehensive cardiac safety analysis. Cancer Chemother. Pharmacol..

[187] Demetri G.D., von Mehren M., Jones R.L., Hensley M.L., Schuetze S.M., Staddon A., Milhen M., Elias A., Ganjoo K., Tawbi H. (2015). Efficacy and Safety of Trabectedin or Dacarbazine for Metastatic Liposarcoma or Leiomyosarcoma After Failure of Conventional Chemotherapy: Results of a Phase III Randomized Multicenter Clinical Trial. J. Clin. Oncol..

[188] Cancer Network Staff FDA Approves Trabectedin (Yondelis) for Advanced Soft-tissue Sarcoma. http://www.cancernetwork.com/sarcoma/fda-approves-trabectedin-yondelis-advanced-soft-tissue-sarcoma.

[189] Cuevas C., Francesch A. (2009). Development of Yondelis (R) (trabectedin, ET-743). A semisynthetic process solves the supply problem. Nat. Prod. Rep..

[190] Carballo J.L., Hernandez-Zanuy A., Naranjo S., Kukurtzu B., Cagide A.G. (1999). Recovery of *Ecteinascidia turbinate* Herman 1880 (Ascidiacea: Perophoridae) populations after different levels of harvesting on a sustainable basis. Bull. Mar. Sci..

[191] Corey E.J., Gin D.Y., Kania R.S. (1996). Enantioselective Total Synthesis of Ecteinascidin 743. J. Am. Chem. Soc..

[192] Martinez E.J., Corey E.J. (2000). A new, more efficient, and effective process for the synthesis of a key pentacyclic intermediate for production of ecteinascidin and phthalascidin antitumor agents. Org. Lett..

[193] Kerr R.G., Miranda N.F. (1995). Biosynthetic Studies of Ecteinascidins in the Marine Tunicate *Ecteinascidia turbinate*. J. Nat. Prod..

[194] Jeedigunta S., Krenisky J.M., Kerr R.G. (2000). Diketopiperazines as Advanced Intermediates in the Biosynthesis of Ecteinascidins. Tetrahedron.

[195] Corey E.J., Gin D.Y. (1996). A Convergent Enantioselective Synthesis of the Tetrahydroisoquinoline Unit in the Spiro Ring of Ecteinascidin 743. Tetrahedron Lett..

[196] Le V.H., Inai M., Williams R.M., Kan T. (2015). Ecteinascidins. A review of the chemistry, biology and clinical utility of potent tetrahydroisoquinoline antitumor antibiotics. Nat. Prod. Rep..

[197] Cuevas C.C., Perez M., Martin M.J., Chicharro C.F., Flores M., Francesch A., Gallego P., Zarzuelo M., de la Calle F., Garcia J. (2000). Synthesis of Ecteinascidin ET-743 and Phthalascidin Pt-650 from Cyanosafracin B. Org. Lett..

[198] Ikeda Y., Matsuki H., Ogawa T., Munakata T. (1983). Safracins, new antitumor antibiotics. 2. Physicochemical properties and chemical structures. J. Antibiot..

[199] Ikeda Y., Idemoto H., Hirayama F., Yamamoto K., Iwao K., Asao T., Munakata T. (1983). Safracins, new antitumor antibiotics. I. Producing organism, fermentation and isolation. J. Antibiot..

[200] Wender P.A., Croatt M.P., Witulski B. (2006). New reactions and step economy: The total synthesis of (±)-salsolene oxide based on the type II transition metal-catalyzed intramolecular [4+4] cycloaddition. Tetrahedron.

[201] Wender P.A., Miller B.L. (2009). Synthesis at the molecular frontier. Nature.

[202] Balczewski P., Mallon M.K.J., Street J.D., Joule J.A. (1990). A synthesis of aaptamine from 6,7-dimethoxy-1-methylisoquinoline. Tetrahedron Lett..

[203] Harada N., Sugioka T., Soutome T., Hiyoshi N., Uda H., Kuriki T. (1995). Synthesis and Absolute Stereochemistry of (+)-Adociaquinones A and B. Tetrahedron Asymmetry.

[204] Han S., Siegel D.S., Morrison K.C., Hergenrother P.J., Movassaghi M. (2013). Synthesis and Anticancer Activity of All Known (–)-Agelastatin Alkaloids. J. Org. Chem..

[205] Murakami N., Sugimoto M., Morita M., Kobayashi M. (2001). Total Synthesis of Agosterol A: An MDR-Modulator from a Marine Sponge. Chem. Eur. J..

[206] Jou G., González I., Albericio F., Lloyd-Williams P., Giralt E. (1997). Total Synthesis of Dehydrodidemnin B. Use of Uronium and Phosphonium Salt Coupling Reagents in Peptide Synthesis in Solution. J. Org. Chem..

[207] Kigoshi H., Ojika M., Ishigaki T., Suenaga K., Mutou T., Sakakura A., Ogawa T., Yamada K. (1994). Total Synthesis of Aplyronine A, a Potent Antitumor Substance of Marine Origin. J. Am. Chem. Soc..

[208] Wang J., Pagenkopf B.L. (2007). First Total Synthesis and Structural Reassignment of (–)-Aplysiallene. Org. Lett..

[209] Yadav J.S., Purnima K.V., Reddy B.V.S., Nagaiah K., Ghamdi A.K. (2011). Total synthesis of cryptophycin-24 (arenastatin A) via Prins cyclization. Tetrahedron Lett..

[210] Sakurai J., Oguchi T., Watanabe K., Abe H., Kanno S., Ishikawa M., Katoh T. (2008). Highly Efficient Total Synthesis of the Marine Natural Products (+)-Avarone, (+)-Avarol, (–)-Neoavarone, (–)-Neoavarol and (+)-Aureol. Chem. Eur. J..

[211] Kotoku N., Tsujita H., Hiramatsu A., Mori C., Koizumi N., Kobayashi M. (2005). Efficient total synthesis of bastadin 6, an anti-angiogenic brominated tyrosine-derived metabolite from marine sponge. Tetrahedron.

[212] Couladouros E.A., Pitsinos E.N., Moutsos V.I., Sarakinos G. (2005). A General Method for the Synthesis of Bastaranes and Isobastaranes: First Total Synthesis of Bastadins 5, 10, 12, 16, 20, and 21. Chem. Eur. J..

[213] Alvarez M., Bros M.A., Gras G., Ajana W., Joule J.A. (1999). Syntheses of Batzelline A, Batzeline B, Isobatzelline A, and Isobatzelline B. Eur. J. Org. Chem..

[214] Manaviazar S., Hale K.J. (2011). Total Synthesis of Bryostatin1: A Short Route. Angew. Chem. Int. Ed..

[215] Keck G.E., Poudel Y.B., Cummins T.J., Rudra A., Covel J.A. (2011). Total Synthesis of Bryostatin 1. J. Am. Chem. Soc..

[216] Prakash K.S., Nagarajan R. (2015). Total synthesis of the marine alkaloids Caulibugulones A and D. Tetrahedron.

[217] Fortner K.C., Kato D., Tanaka Y., Shair M.D. (2010). Enantioselective Synthesis of (+)-Cephalostatin 1. J. Am. Chem. Soc..

[218] Forestieri R., Donohue E., Balgi A., Roberge M., Andersen R.J. (2013). Synthesis of Clionamine B, an Autophagy Stimulating Aminosteroid Isolated from the Sponge *Cliona celata*. Org. Lett..

[219] Shi J., Manolikakes G., Yeh C.-H., Guerrero C.A., Shenvi R.A., Shigehisa H., Baran P.S. (2011). Scalable Synthesis of Cortistatin A and Related Structures. J. Am. Chem. Soc..

[220] Dunetz J., Julian L.D., Newcom J.S., Roush W.R. (2008). Total Syntheses of (+)-Tedanolide and (+)-13-Deoxytedanolide. J. Am. Chem. Soc..

[221] Knowles R.R., Carpenter J., Blakey S.B., Kayano A., Mangion I.K., Sinz C.J., MacMillan D.W.C. (2011). Total Synthesis of Diazonamide A. Chem. Sci..

[222] Sumii Y., Kotoku N., Fukuda A., Kawachi T., Sumii Y., Arai M., Kobayashi M. (2015). Enantioselective synthesis of dictyoceratin-A (smenospondiol) and -C, hypoxia-selective growth inhibitors from marine sponge. Bioorg. Med. Chem..

[223] Ho S., Bucher C., Leighton J.L. (2013). A Highly Step-Economical Synthesis of Dictyostatin. Angew. Chem. Int. Ed..

[224] Li W.-R., Ewing W.R., Harris B.D., Joullié M.M. (1990). Total Synthesis and Structural Investigations of Didemnins A, B, and C. J. Am. Chem. Soc..

[225] Yu Z., Ely R.J., Morken J.P. (2014). Synthesis of (+)-Discodermolide by Catalytic Stereoselective Borylation Reactions. Angew. Chem. Int. Ed..

[226] Mordant C., Reymond S., Tone H., Lavergne D., Touati R., Hassine B.B., Ratovelomanana-Vidal V., Genet J.-P. (2007). Total Synthesis of dolastatin 10 through ruthenium-catalyzed asymmetric hydrogenations. Tetrahedron.

[227] Akaji K., Hayashi Y., Kiso Y., Kuriyama N. (1999). Convergent Synthesis of Dolastatin 15 by Solid Phase Coupling of an *N*-Methylamino Acid. J. Org. Chem..

[228] Chen X.-T., Bhattacharya S.K., Zhou B., Gutteridge C.E., Pettus T.R.R., Danishefsky S.J. (1999). The Total Synthesis of Eleutherobin. J. Am. Chem. Soc..

[229] Bharate S.B., Manda S., Joshi P., Singh B., Vishwakarma R.A. (2012). Total synthesis and anti-cholinesterase activity of marine-derived bis-indole alkaloid fascaplysin. Med. Chem. Commun..

[230] Kotoku N., Fujioka S., Nakata C., Yamada M., Sumii Y., Kawachi T., Arai M., Kobayashi M. (2011). Concise synthesis and structure-activity relationship of furospinosulin-1, a hypoxia-selective growth inhibitor from marine sponge. Tetrahedron.

[231] Boukouvalas J., Albert V. (2011). Synthesis of the Hypoxic Signaling Inhibitor Furospongolide. Syn. Lett..

[232] White J.D., Amedio J.C. (1989). Total Synthesis of Geodiamolide A, a Novel Cyclodepsipeptide of Marine Origin. J. Org. Chem..

[233] Hirai Y., Yokota K., Yamzaki T., Momose T. (1990). A Total Synthesis of (+)-Geodiamolides A and B, the Novel Cyclodesipeptides. Heterocycles.

[234] Fung S.-Y., Sofiyev V., Schneiderman J., Hirschfeld A.F., Victor R.E., Woods K., Piotrowski J.S., Deshpande R., Li S.C., de Voogd N.J. (2014). Unbiased Screening of Marine Sponge Extracts for Anti-inflammatory Agents Combined with Chemical Genomics Identifies Girolline as an Inhibitor of Protein Synthesis. ACS Chem. Biol..

[235] Aicher T.D., Buszek K.R., Fang F.G., Forsyth C.J., Jung S.H., Kishi Y., Matelich M.C., Scola P.M., Spero D.M., Yoon S.K. (1992). Total Synthesis of Halichondrin B and Norbalichondrin B. J. Am. Chem. Soc..

[236] Somaiah R., Ravindar K., Cencic R., Pelletier J., Deslongchamps P. (2014). Synthesis of the Antiproliferative Agent Hippuristanol and Its Analogues from Hydrocortisone via Hg(II)-Catalyzed Spiroketalization: Structure-Activity Relationship. J. Med. Chem..

[237] Shearman J.W., Myers R.M., Beale T.M., Brenton J.D., Ley S.V. (2010). Total syntheses of the bromotyrosine-derived natural products ianthelline, 5-bromoverongamine and JBIR-44. Tetrahedron Lett..

[238] Fürstner A., Nevado C., Waser M., Tremblay M., Chevrier C., Teplý F., Aïssa C., Moulin E., Müller O. (2007). Total Synthesis of Iejimalide A-D and Assessment of the Remarkable Actin-Depolymerizing Capacity of These Polyene Macrolides. J. Am. Chem. Soc..

[239] Ling T., Poupon E., Rueden E.J., Theodorakis E.A. (2002). Synthesis of (–)-Ilimaquinone via a Radical Decarboxylation and Quinone Addition Reaction. Org. Lett..

[240] Uesugi S., Watanabe T., Imaizumi T., Ota Y., Yoshida K., Ebisu H., Chinen T., Nagumo Y., Shibuya M., Kanoh N. (2015). Total Synthesis and Biological Evaluation of Irciniastatin A (a.k.a. Psymberin) and Irciniastatin B. J. Org. Chem..

[241] Dobbs A.P., Venturelli A., Butler L.A., Parker R.J. (2005). First Total Synthesis of the Irciniasulfonic Acids. Syn. Lett..

[242] López-Macia A., Jiménez J.C., Royo M., Giralt E., Albericio F. (2001). Synthesis and Structure Determination of Kahalalide F. J. Am. Chem. Soc..

[243] Li Q., Jiang J., Fan A., Cui Y., Jia Y. (2011). Total Synthesis of Lamellarins D, H, and R and Ningalin B. Org. Lett..

[244] Trost B.M., Stivala C.E., Hull K.L., Huang A., Fandrick D.R. (2014). A Concise Synthesis of (–)-Lasonolide A. J. Am. Chem. Soc..

[245] Fürstner A., De Souza D., Turet L., Fenster M.D.B., Parra-Rapado L., Wirtz C., Mynott R., Lehmann C.W. (2007). Total Syntheses of the Actin-Binding Macrolides Latrunculin A, B, C, M, S and 16-*epi*-Latrunculin B. Chem. Eur. J..

[246] Trost B.M., Amans D., Seganish W.M., Chung C.K. (2012). Total Synthesis of Laulimalide: Assembly of the Fragments and Completion of the Synthesis of the Natural Product and Potent Analogue. Chem. Eur. J..

[247] Boukouvalas J., Robichaud J., Maltais F. (2006). A Unified Strategy for the Regiospecific Assembly of Homoallyl-Substituted Butenolides and y-Hydroxybutenolides: First Synthesis of Luffariellolide. Synlett.

[248] Oshiyama T., Satoh T., Okano K., Tokuyama H. (2012). Total Synthesis of makaluvamine A/D, damirone B, batzelline C, makaluvone, and isobatzelline C featuring one-pot benzyne-mediated cyclization-functionalization. Tetrahedron.

[249] Jakubec P., Hawkins A., Felzmann W., Dixon D.J. (2012). Total Synthesis of Manzamine A and Related Alkaloids. J. Am. Chem. Soc..

[250] Fresneda P.M., Molina P., Bleda J.A. (2001). Synthesis of the indole alkaloids meridianins from tunicate *Aplidium meridianum*. Tetrahedron.

[251] Chandrasekhar S., Sultana S.S. (2006). Stereoselective synthesis of the C1-C20 segment of the microsclerodermins A and B. Tetrahedron Lett..

[252] Shi Y., Pierce J.G. (2015). Synthesis of the 5,6-Dihydroxymorpholin-3-one Fragment of Monanchocidin A. Org. Lett..

[253] Sohn J.H., Waizumi N., Zhong M., Rawal V.H. (2005). Total synthesis of mycalamide A. J. Am. Chem. Soc..

[254] Wang L., Hale K.J. (2015). Total Synthesis of the Potent HIF-1 Inhibitory Antitumor Natural Product, (8*R*)-Mycothiazole, via Baldwin-Lee CsF/CuI sp^3^-sp^2^-Stille Cross-Coupling. Confirmation of the Crews Reassignment. Org. Lett..

[255] Pérez M., del Poz C., Reyes F., Rodríguez A., Francesch A., Echavarren A.M., Cuevas C. (2004). Total Synthesis of Natural Myriaporones. Angew. Chem. Int. Ed..

[256] Gao S., Wang Q., Chen C. (2009). Synthesis and Structure Revision of Nakiterpiosin. J. Am. Chem. Soc..

[257] Li L., Abraham A.D., Zhou Q., Ali H., O’Brien J.V., Hamill B.D., Arcaroli J.J., Messersmith W.A., LaBarbera D.V. (2014). An Improved High Yield Total Synthesis and Cytotoxicity Study of the Marine Alkaloid Neoamphimedine: An ATP-Competitive Inhibitor of Topoisomerase Iiα and Potent Anticancer Agent. Mar. Drugs..

[258] Liu H., Boudreau M.A., Zheng J., Whittal R.M., Austin P., Roskelley C.D., Roberge M., Andersen R.J., Vederas J.C. (2010). Chemical Synthesis and Biological Activity of the Neopetrosiamides and Their Analogues: Revision of Disulfide Bond Connectivity. J. Am. Chem. Soc..

[259] Boger D.L., Boyce C.W., Labroli M.A., Sehon C.A., Jin Q. (1999). Total Syntheses of Ningalin A, Lamellarin O, Lukianol A, and Permethyl Storniamide A Utilizing Heterocyclic Azadiene Diels-Alder Reactions. J. Am. Chem. Soc..

[260] Hong C.Y., Kishi Y. (1991). Total Synthesis of Onnamide A. J. Am. Chem. Soc..

[261] Dhand V., Chang S., Britton R. (2013). Total Synthesis of the Cytotoxic Anhydrophytosphingosine Pachastrissamine (Jaspine B). J. Org. Chem..

[262] Seiple I.B., Su S., Young I.S., Nakamura A., Yamaguchi J., Jørgensen L., Rodriguez R.A., O’Malley D.P., Gaich T., Köck M. (2011). Enantioselective Total Syntheses of (–)-Palau’amine, (–)-Axinellamines, and (–)-Massadines. J. Am. Chem. Soc..

[263] Pattenden G., Critcher D.J., Remuiñán M. (2004). Total synthesis of (-)-pateamine A, a novel immunosuppressive agent from Mycale sp^1^. Can. J. Chem..

[264] Trost B.M., Michaelis D.J., Malhotra S. (2013). Total Synthesis of (–)-18-*epi*-Peloruside A: An Alkyne Linchpin Strategy. Org. Lett..

[265] Martín M.J., Coello L., Fernández R., Reyes F., Rodríguez A., Murcia C., Garranzo M., Mateo C., Sánchez-Sancho F., Bueno S. (2013). Isolation and First Total Synthesis of PM050489 and PM060184, Two New Marine Anticancer Compounds. J. Am. Chem. Soc..

[266] Hong S., Shin Y., Jung M., Ha M.W., Park Y., Lee Y.-J., Shin J., Oh K.B., Lee S.K., Park H.-G. (2015). Efficient synthesis and biological activity of Psammaplin A and its analogues as antitumor agents. Eur. J. Med. Chem..

[267] Georgiades S.N., Clardy J. (2005). Total Synthesis of Psammaplysenes A and B, Naturally Occurring Inhibitors of FOXO1a Nuclear Export. Org. Lett..

[268] Zhu J., Wu Y.-C. (2009). Asymmetric Total Syntheses of (–)-Renieramycin M and G and (–)-Jorumycin Using Aziridine as a Lynchpin. Org. Lett..

[269] Herb C., Bayer A., Maier M.E. (2004). Total Synthesis of Salicylihalamides A and B. Chem. Eur. J..

[270] Nicolaou K.C., Xu J.Y., Kim S., Pfefferkorn J., Ohshima T., Vourloumis D., Hosokawa S. (1998). Total Synthesis of Sarcodictyins A and B. J. Am. Chem. Soc..

[271] Ma Z., Wang X., Wang X., Rodriguez R.A., Moore C.E., Gao S., Tan X., Ma Y., Rheingold A.L., Baran P.S. (2014). Asymmetric syntheses of sceptrin and massadine and evidence for biosynthetic enantiodivergence. Science.

[272] Shinkre B.A., Velu S.E. (2007). Total Synthesis of Secobatzelline B. Synth. Commun..

[273] Ghosal P., Shaw A.K. (2010). An efficient total synthesis of the anticancer agent (+)-spisulosine (ES-285) from Garner’s aldehyde. Tetrahedron Lett..

[274] Smith A.B., Sfouggatakis C., Risatti C.A., Sperry J.B., Zhu W., Doughty V.A., Tomioka T., Gotchev D.B., Bennett C.S., Sakamoto S. (2009). Spongipyran synthetic studies. Evolution of a scalable total synthesis of (+)-spongistatin 1. Tetrahedron.

[275] Shearman J.W., Myers R.M., Brenton J.D., Ley S.V. (2011). Total Syntheses of subereamollines A and B. Org. Biomol. Chem..

[276] Kawagishi F., Toma T., Inui T., Yokoshima S., Fukuyama T. (2013). Total synthesis of ecteinascidin 743. J. Am. Chem. Soc..

[277] Anderson R.J., Morris J.C. (2001). Total synthesis of variolin B. Tetrahedron Lett..

[278] Zurwerra D., Glaus F., Betschart L., Schuster J., Gertsch J., Ganci W., Altmann K.-H. (2012). Total Synthesis of (–)-Zampanolide and Structure-Activity Relationship Studies on (–)-Dactylolide Derivatives. Chem. Eur. J..

[279] Perry N.B., Blunt J.W., Munro M.H., Pannell L.K. (1988). Mycalamide A, an antiviral compound from a New Zealand sponge of the genus *Mycale*. J. Am. Chem. Soc..

[280] Burres N.S., Clement J.J. (1989). Antitumor activity and mechanism of action of the novel marine natural products mycalamide-A and -B and onnamide. Cancer Res..

[281] Gürel G., Blaha G., Steitz T.A., Moore P.B. (2009). Structures of triacetyloleandomycin and mycalamide A bind to the large ribosomal subunit of *Haloarcula marismortui*. Antimicrob. Agents Chemother..

[282] Simpson J.S., Garson M.J., Blunt J.W., Munro M.H.G., Hooper J.N.A. (2000). Mycalamides C and D, cytotoxic compounds from the marine sponge *Stylinos* n. species. J. Nat. Prod..

[283] Venturi V., Davies C., Singh A.J., Matthews J.H., Bellows D.S., Northcote P.T., Keyzers R.A., Teesdale-Spittle P.H. (2012). The protein synthesis inhibitors mycalamides A and E have limited susceptibility toward the drug efflux network. J. Biochem. Mol. Toxicol..

[284] Dyshlovoy S.A., Fedorov S.N., Kalinovsky A.I., Shubina L.K., Bokemeyer C., Stonik V.A., Honecker F. (2012). Mycalamide A shows cytotoxic properties and prevents EGF-induced neoplastic transformation through inhibition of nuclear factors. Mar. Drugs.

[285] Pettit G.R., Cichacz Z.A., Gao F., Herald C.L., Boyd M.R., Schmidt J.M., Hooper J.N. (1993). Isolation and structure of spongistatin 1. J. Org. Chem..

[286] Kobayashi M., Aoki S., Sakai H., Kawazoe K., Kihara N., Sasaki T., Kitagawa I. (1993). Altohyrtin A, a potent antitumor macrolide from the Okinawan marine sponge *Hyrtios altum*. Tetrahedron Lett..

[287] Bai R., Taylor G.F., Cichacz Z.A., Herald C.L., Kepler J.A., Pettit G.R., Hamel E. (1995). The spongistatins, potently cytotoxic inhibitors of tubulin polymerization, bind in a distinct region of the vinca domain. Biochemistry.

[288] Rothmeier A.S., Ischenko I., Joore J., Garczarczyk D., Fürst R., Bruns C.J., Vollmar A.M., Zahler S. (2009). Investigation of the marine compound spongistatin 1 links the inhibition of PKCα translocation to nonmitotic effects of tubulin antagonism in angiogenesis. FASEB J..

[289] Schneiders U.M., Schyschka L., Rudy A., Vollmar A.M. (2009). BH3-only proteins Mcl-1 and Bim as well as endonuclease G are targeted in spongistatin 1-induced apoptosis in breast cancer cells. Mol. Cancer Ther..

[290] Su J.Y., Meng Y.H., Zeng L.M., Fu X., Schmitz F.J. (1994). Stellettin A, a new triterpenoid pigment from the marine sponge *Stelletta tenuis*. J. Nat. Prod..

[291] Guzii A.G., Makarieva T.N., Denisenko V.A., Dmitrenok P.S., Kuzmich A.S., Dyshlovoy S.A., Krasokhin V.B., Stonik V.A. (2010). Monanchocidin: A new apoptosis-inducing polycyclic guanidine alkaloid from the marine sponge *Monanchera pulchra*. Org. Lett..

[292] Liu W.K., Ling Y.H., Cheung F.W., Che C.T. (2012). Stellettin A induces endoplasmic reticulum stress in murine B16 melanoma cells. J. Nat. Prod..

[293] Dyshlovoy S.A., Hauschild J., Amann K., Tabakmakher K.M., Venz S., Walther R., Guzii A.G., Makarieva T.N., Shubina L.K., Fedorov S.N. (2015). Marine alkaloid monanchocidin A overcomes drug resistance by induction of autophagy and lysosomal membrane permeabilization. Oncotarget.

[294] McKee T.C., Bokesch H.R., McCormick J.L., Rashid M.A., Spielvogel D., Gustafson K.R., Alavanja M.M., Boyd M.R. (1997). Isolation and characterization of new anti-HIV and cytotoxic leads from plants, marine, and microbial organisms. J. Nat. Prod..

[295] McCormick J.L., McKee T.C., Cardellina J.H., Leid M., Boyd M.R. (1996). Cytotoxic triterpenes from a marine sponge, *Stelletta* sp.. J. Nat. Prod..

[296] Lv F., Deng Z., Li J., Fu H., van Soest R.W.M., Proksch P., Lin W. (2004). Isomalabaricane-type compounds from the marine sponge *Rhabdastrella* aff. *distincta*. J. Nat. Prod..

[297] Makarieva T.N., Tabakmaher K.M., Guzii A.G., Denisenko V.A., Dmitrenok P.S., Shubina L.K., Kuzmich A.S., Lee H.-S., Stonik V.A. (2011). Monanchocidins B–E: Polycyclic guanidine alkaloids with potent antileukemic activities from the sponge *Monanchora pulchra*. J. Nat. Prod..

[298] Mudit M., Khanfar M., Muralidharan A., Thomas S., Shah G.V., van Soest R.W., El Sayed K.A. (2009). Discovery, design, and synthesis of anti-metastatic lead phenylmethylene hydantoins inspired by marine natural products. Bioorg. Med. Chem..

[299] Shah G.V., Muralidharan A., Thomas S., Gokulgandhi M., Mudit M., Khanfar M., El Sayed K. (2009). Identification of a small molecule class to enhance cell-cell adhesion and attenuate prostate tumor growth and metastasis. Mol. Cancer Ther..

[300] Sallam A.A., Mohyeldin M.M., Foudah A.I., Akl M.R., Nazzal S., Meyer S.A., Liu Y.Y., El Sayed K.A. (2014). Marine natural products-inspired phenylmethylene hydantoins with potent *in vitro* and *in vivo* antitumor activities via suppression of Brk and FAK signaling. Org. Biomol. Chem..

[301] Jin J.O., Shastina V.V., Shin S.W., Xu Q., Park J.-I., Rasskazov V.A., Avilov S.A., Fedorov S.N., Stonik V.A., Kwak J.-Y. (2009). Differential effects of triterpene glycosides, frondoside A and cucumarioside A2–2 isolated from sea cucumbers on caspase activation and apoptosis of human leukemia cells. FEBS Lett..

[302] Janakiram N.B., Mohammed A., Zhang Y., Choi C.-I., Woodward C., Collin P., Steele V.E., Rao C.V. (2010). Chemopreventive effects of Frondanol A5, a *Cucumaria frondosa* extract, against rat colon carcinogenesis and inhibition of human colon cancer cell growth. Cancer Prev. Res..

[303] Ma X., Kundu N., Collin P.D., Goloubeva O., Fulton A.M. (2012). Frondoside A inhibits breast cancer metastasis and antagonizes prostaglandin E receptors EP4 and EP2. Breast Cancer Res. Treat..

[304] Park S.Y., Kim Y.H., Kim Y., Lee S.-J. (2012). Frondoside A has an anti-invasive effect by inhibiting TPA-induced MMP-9 activation via NF-κB and AP-1 signaling in human breast cancer cells. Int. J. Oncol..

[305] Attoub S., Arafat K., Gélaude A., Al Sultan M.A., Bracke M., Collin P., Takahashi T., Adrian T.E., de Wever O. (2013). Frondoside A suppressive effects on lung cancer survival, tumor growth, angiogenesis, invasion, and metastasis. PLoS ONE.

[306] Avilov S.A., Kalinin V.I., Prozdova O.A., Kalinovskii A.I., Stonik V.A., Gudimova E.N. (1993). Triterpene glycosides from the holothurian *Cucumaria frondosa*. Chem. Nat. Comp..

[307] Silchenko A.S., Avilov S.A., Kalinin V.I., Kalinovsky A.I., Dmitrenok P.S., Fedorov S.N., Stepanov V.G., Dong Z., Stonik V.A. (2008). Constituents of the sea cucumber *Cucumaria okhotensis*. Structures of okhotosides B_1_-B_3_ and cytotoxic activities of some glycosides from this species. J. Nat. Prod..

[308] Floss H.G. (2006). Combinatorial biosynthesis—Potential and problems. J. Biotechnol..

[309] Menzella H.G., Reeves C.D. (2007). Combinatorial biosynthesis for drug development. Curr. Opin. Microbiol..

[310] Kennedy J. (2008). Mutasynthesis, chemobiosynthesis, and back to semi-synthesis: Combining synthetic chemistry and biosynthetic engineering for diversifying natural products. Nat. Prod. Rep..

[311] Kirschning A., Hahn F. (2012). Merging chemical synthesis and biosynthesis: A new chapter in the total synthesis of natural products and natural product libraries. Angew. Chem. Int. Ed. Engl..

[312] Regentin R., Kennedy J., Wu N., Carney J.R., Licari P., Galazzo J., Desai R. (2004). Precursor-directed biosynthesis of novel triketide lactones. Biotechnol. Prog..

[313] Gerwick W.H., Moore B.S. (2012). Lessons from the past and charting the future of marine natural products drug discovery and chemical biology. Chem. Biol..

[314] Cragg G.M., Newman D.J. (2013). Natural products: A continuing source of novel drug leads. Biochim. Biophys. Acta.

[315] Li R. (2016). Marinopyrroles: Unique drug discoveries based on marine natural products. Med. Res. Rev..

[316] Vinothkumar S., Parameswaran P.S. (2013). Recent advances in marine drug research. Biotechnol. Adv..

